# Lineage-specific genes are clustered with HET-domain genes and respond to environmental and genetic manipulations regulating reproduction in *Neurospora*

**DOI:** 10.1371/journal.pgen.1011019

**Published:** 2023-11-07

**Authors:** Zheng Wang, Yen-Wen Wang, Takao Kasuga, Francesc Lopez-Giraldez, Yang Zhang, Zhang Zhang, Yaning Wang, Caihong Dong, Anita Sil, Frances Trail, Oded Yarden, Jeffrey P. Townsend

**Affiliations:** 1 Department of Biostatistics, Yale School of Public Health, New Haven, Connecticut, United States of America; 2 College of Biological Sciences, University of California, Davis, California, United States of America; 3 Yale Center for Genomic Analysis, New Haven, Connecticut, United States of America; 4 National Genomics Data Center, Beijing Institute of Genomics, Chinese Academy of Sciences, Beijing, China; 5 Institute of Microbiology, Chinese Academy of Sciences, Beijing, China; 6 Department of Microbiology and Immunology, University of California, San Francisco, California, United States of America; 7 Department of Plant, Soil and Microbial Sciences, Michigan State University, East Lansing, Michigan, United States of America; 8 Department of Plant Pathology and Microbiology, The Robert H. Smith Faculty of Agriculture, Food and Environment, The Hebrew University of Jerusalem, Rehovot, Israel; 9 Department of Ecology and Evolutionary Biology, Program in Microbiology, and Program in Computational Biology and Bioinformatics, Yale University, New Haven, Connecticut, United States of America; Australian National University Research School of Biology, AUSTRALIA

## Abstract

Lineage-specific genes (LSGs) have long been postulated to play roles in the establishment of genetic barriers to intercrossing and speciation. In the genome of *Neurospora crassa*, most of the 670 *Neurospora* LSGs that are aggregated adjacent to the telomeres are clustered with 61% of the HET-domain genes, some of which regulate self-recognition and define vegetative incompatibility groups. In contrast, the LSG-encoding proteins possess few to no domains that would help to identify potential functional roles. Possible functional roles of LSGs were further assessed by performing transcriptomic profiling in genetic mutants and in response to environmental alterations, as well as examining gene knockouts for phenotypes. Among the 342 LSGs that are dynamically expressed during both asexual and sexual phases, 64% were detectable on unusual carbon sources such as furfural, a wildfire-produced chemical that is a strong inducer of sexual development, and the structurally-related furan 5-hydroxymethyl furfural (HMF). Expression of a significant portion of the LSGs was sensitive to light and temperature, factors that also regulate the switch from asexual to sexual reproduction. Furthermore, expression of the LSGs was significantly affected in the knockouts of *adv-1* and *pp-1* that regulate hyphal communication, and expression of more than one quarter of the LSGs was affected by perturbation of the mating locus. These observations encouraged further investigation of the roles of clustered lineage-specific and HET-domain genes in ecology and reproduction regulation in *Neurospora*, especially the regulation of the switch from the asexual growth to sexual reproduction, in response to dramatic environmental conditions changes.

## Author summary

A portion of genes within sequenced genomes are lineage-specific. These lineage-specific genes (LSGs) lack evolutionary histories tracking them to ancestors within the genomes of other lineages. Accordingly, they are often classified as new or de novo genes and have long been postulated to play roles in the speciation. Here 670 *Neurospora* LSGs are reported, most of which are aggregate adjacent to the telomeres and are clustered along with “HET-domain” genes, some of which perform functions in the regulation of self-recognition. In contrast, the LSG-encoding proteins possess few to no domains that would help to identify potential functional roles. We assessed possible functional roles of LSGs by performing transcriptomic profiling in genetic mutants and in distinct environmental conditions, as well as examining gene knockouts for phenotypes. Many LSGs are actively regulated during both asexual and sexual reproduction in response to carbon-resource, light, and temperature-based environmental factors. Furthermore, expression of the LSGs was reported to be significantly affected by perturbation of the genes *adv-1*, *pp-1*, and a mating locus that regulates hyphal communication and initiation of sexual reproduction in the fungus. These observations encouraged further investigation of the roles of clustered LSGs and HET-domain genes in ecology and reproduction regulation in *Neurospora*.

## Introduction

Since the emergence of life, molecular evolution has contributed to the accumulation of novel and diverse features in all kinds of organisms. Two fundamental components of that molecular evolutionary novelty are new genes and novel gene functions, which have long been considered to be emergent properties of gene duplication and rearrangement. Nevertheless, genomes often harbor numerous orphan genes or lineage specific genes (LSGs)—novel genes that have no homologues in distantly- or closely-related lineages and that cannot be tracked to ancestral lineages. These LSGs, including de novo genes that evolve from previous non-coding DNA and non-genic elements [1], manifest in large numbers across a diversity of organisms, such that they represent nearly one-third of the genes in all genomes, including phages, archaea, bacteria, and eukaryotic organisms [2]. Three key challenges have thus been the focus of studies of LSGs: how to identify LSGs, how to track their evolutionary histories, and perhaps most importantly, how these genes are integrated into pre-existing gene interaction networks [3].

Accurate identification of LSGs can, at times, be difficult. They originate via three evolutionary processes: (1) rapid gene evolution, which is refractory to tracking homology on the basis of sequence conservation; (2) intragenomic gene loss and gain or horizontal gene transfer, which can convey higher fitness in response to genetic/environmental changes; and (3) accumulated mutations that establish novel function, evolving slowly but long enough on an independent lineage that the gene phylogeny cannot be tracked back to its distant ancestor or to related lineages [4–6].

Studies of the genomic characteristics of LSGs in several model organisms have revealed the likely origins of LSGs from gene duplication, non-coding sequences, as well as fast evolution at conserved genomic positions. One example supporting the frequency of origin by rapid divergence after gene duplication and rearrangement can be found in yeast, where the presence of 55% to 73% percent of the LSGs can be explained by sufficient divergence from sister species [6]. Some lineage-specific protein-coding genes might have directly evolved from non-coding regions in the genome [7], as has been described in the tests of fruit flies [8,9]. One hundred seventy-five *de novo* genes in Asian rice corresponded to recognizable non-genic sequences in closely related species [10]. These investigations using model organisms confirmed unique characteristics of these *de novo* genes, making good frameworks for investigating LSGs in other species [11]. However, linkages between these revealed genomic characteristics and the integrative functions of LSGs remain unclear. Therefore, systematic approaches that combine study of comparative genomics with functional assays of gene expression and gene-perturbation phenotypes using well-established model systems are critical to integrate the investigation of the LSGs’ origination and function.

The most frequently used approach to identify LSGs is using phylostratigraphy [12,13]. Precise identification of the origins of *de novo* genes using a phylostratigraphic approach is critically dependent on accurate gene annotation and extensive comparison among proper representative genomes [14]. It is difficult to distinguish whether genes with no homologues in closely related lineages are true LSGs as opposed to lacking homologues in closely related lineages that have few genomes available. An alternative to phylostratigraphy is gene synteny, which compares each gene’s position relative to its neighbors. A recent study suggested that if the neighbors of a gene are in a conserved order in other species, then the gene is likely to correspond to whatever is at the orthologous position in the other species as well—even if the sequences do not match [15].

LSGs are naturally thought to be important to species- or genus-level adaptations of development to taxon-specific ecology. Identification of LSGs, including *de novo* protein-coding genes, need to be further verified with a systematic approach focusing on possible functional novelty and genetic signals that may be associated with such a novelty [3]. Systematic assessment of the putative LSG function can track their behaviors during the growth and development and verify their possible roles by examination of corresponding knockdown or knockout phenotypes [3]. The well-annotated model species in the genus *Neurospora*, *N*. *crassa*, *N*. *tetrasperma* and *N*. *discreta* of the class Sordariomycetes, provide a set of three closely-related genomes enabling investigation of possible genetic novelties associated with recent and rapid ecological divergences [16], such as responses to nutrients and other environmental factors and developmental divergences in reproduction. *Neurospora* species are highly adapted to the postfire environment, capable of fast asexual growth and reproduction on simple nutrients and have long been genetic models for eukaryotic metabolic regulation and for mating, meiosis and morphological development during reproduction [17–19]. Comparing representative genomes in prokaryotes, plants & animals, Basidiomycota, major lineages of Ascomycota, and *Chaetomium globosum*, which is closely related to *N*. *crassa*, 2219 orphan genes were identified in *N*. *crassa* by phylostratigraphic analysis, which determines the age of origin of every gene in a genome [20]. In the past decade, many more fungal genomes have been sequenced. Their sequence, along with the advances in genome sequencing and annotation techniques [21–23], provide a more inclusive comparison for identifying lineage specific genes. Therefore, to understand how important the roles that LSGs play in genome-wide regulation during the whole life history, we investigated possible roles of LSGs in environmental responses and reproduction regulation in *Neurospora*.

We also observed that several LSGs are also annotated HET-domain genes. Several *het* genes regulate allorecognition during vegetative growth, and only individuals with compatibility at all of their *het* loci can fuse and simply expand their colonies [24]. Some HET-domain genes have pleiotropic effects in sexual development in some fungal species and play direct roles in reproductive isolation and speciation within sympatry [25]. There were 69 HET-domain genes reported (of which 68 were mapped to the original genome-sequenced strain of *N*. *crassa*) [24]. However, functions of HET-domain genes remain largely unknown, and some genes without the HET-domain also reported in allorecognition and programmed cell death in *Neurospora* [26,27]. Chromosomal locations that are itinerant over evolutionary time suggest the merits of integrative investigations of both gene groups.

## Results

### Summary

In this study, we identified and verified 670 lineage-specific genes (LSGs) in *Neurospora crassa* via BLAST search against inclusive representative genomes. Orphan genes predicted in a previously published phylostratigraphic study of *Neurospora* were used as a preliminary list, which was based on a limited set of representative genomes [20]. Using two clustering approaches, we discovered that over 60% of the 670 LSGs formed clusters in the telomere regions and clustered with the HET-domain genes. However, most of the LSGs are not functionally annotated (e.g., with gene ontology terms). Therefore, to assess the possible functional roles of the LSGs, we analyzed genome-wide gene expression data on *N*. *crassa* growing on distinct media at different stages of life history and under different light and temperature conditions. We observed that nearly half of the LSGs were actively expressed during asexual and sexual growth. A substantial number (291) LSGs were induced by the presence of furfural. 158 LSGs were exclusively expressed in furfural cultures, in contrast to only 17 LSGs that were exclusively expressed in cultures on media supplied with common simple carbohydrates. We also reported a significant portion of the LSGs being turned on or off by the changes of light exposure and temperature, conditions that are critical for *N*. *crassa* asexual and sexual reproduction. We further examined expression of the lineage-specific and HET-domain genes in knockouts of two transcription factors, *adv-1* and *pp-1*. Both transcription factors play multiple roles in asexual and sexual development in *N*. *crassa*. In addition, we sequenced and analyzed genome-wide gene expression in a loss-function mutant at the mating locus *mat 1-2-1* in a *mat a* strain during the crossing. The LSGs were more likely to be affected by gene-manipulation than other genes, compared with other non-LSG genes and HET-domain genes. They were more likely to be turned on or turned off completely, rather than being turned up or down slightly. Finally, we examined asexual and sexual growth phenotypes for 367 available KO strains of the *Neurospora* LSGs. We identified two LSGs with abortive sexual reproduction, and several LSGs with minor phenotypes in response to high temperature or mycelium morphology.

### 670 LSGs were identified in Neurospora genomes

**Fig 1:**
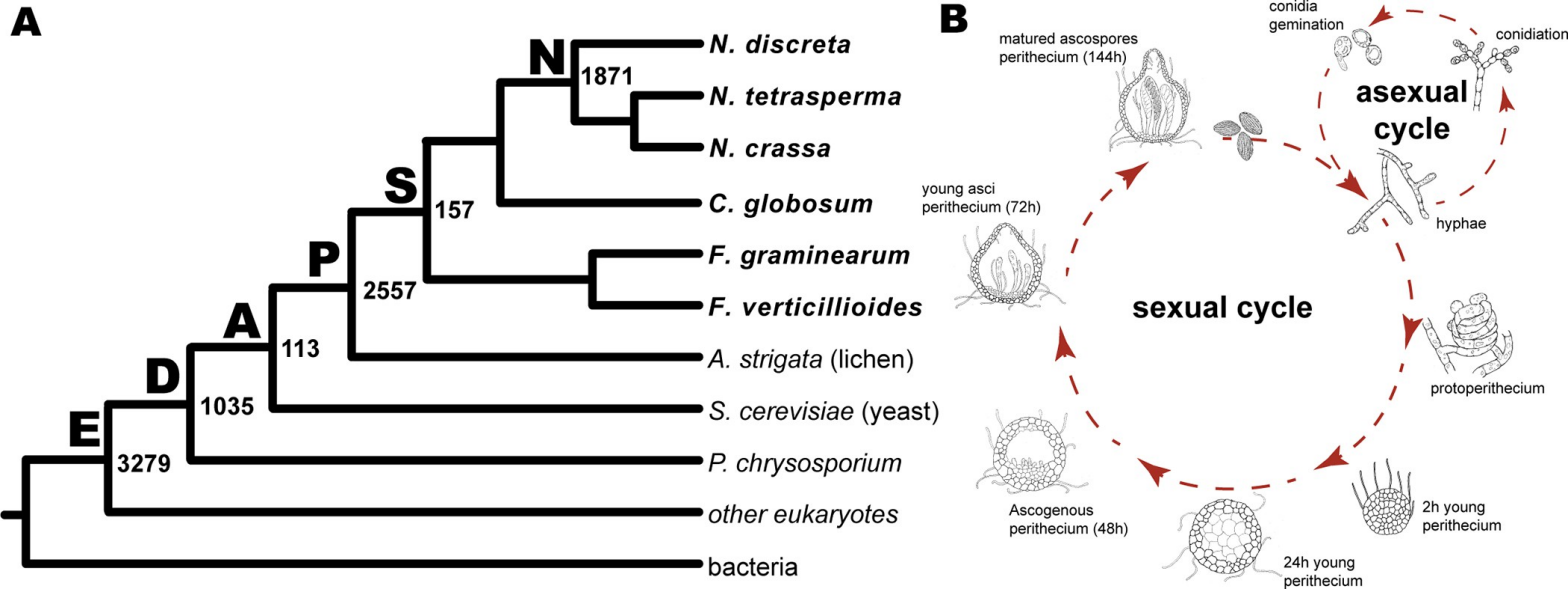
Systematics of *Neurospora* lineage-specific genes (LSGs) and potential roles in fungal growth and development. (**A**) Genomic phylostratigraphy of lineage-specificity classifications of predicted protein- coding genes (enumerated at ancestral nodes: 3279 **E**ukaryote-core, 1035 **D**ikarya-core, 113 **A**scomycota-core, 2557 **P**ezizomycotina-specific, 157 **S**ordariomycetes-specific, and 1871 **N**eurospora- specific) that are present within the *N*. *crassa* genome; (**B**) Life history of *N*. *crassa*. These developmental processes have been transcriptionally profiled [dashed red arrows; 28].

We defined *Neurospora* lineage-specific genes (LSGs) as *N*. *crassa* genes exhibiting homology to only to genes found in sequences from species within the genus *Neurospora* [12]. To identify *Neurospora* LSGs, phylostratigraphy was performed on representative taxa for major fungal lineages and on several non-fungal reference genomes: 1872 *N*. *crassa* genes were identified as putative LSGs (**Fig 1 and S1 Table**). Within these 1872 *N*. *crassa* genes, a total of 695 genes are shared between the genomes of *Neurospora* and the sole species *S*. *macrospora* in the sister genus (**S1 Table**). Further reciprocal-BLAST searches were made for the 1872 genes against available Sordariomycetes genomes, including genomes of *Neurospora* closely related species in *Podospora*, *Pyricularia* and *Ophiostoma* as well as species within the genus including *N*. *tetrasperma* and *N*. *discreta* at the National Center for Biotechnology (NCBI) and FungiDB genome database. We identified 670 genes that are *N*. *crassa* lineage-specific genes (LSGs) (**S1 Fig** and **S1 Table**). There are 7400 single-copy orthologs shared among the genomes of *N*. *crassa*, *N*. *discreta*, and *N*. *tetrasperma* (**S1A Fig**). Among the 670 LSGs identified in the *N*. *crassa* genome, 241 are unique to *N*. *crassa* and 405 are shared between *N*. *crassa* and *N*. *tetrasperma* with 248 showing no orthologs in *N*. *discreta*. 181 *Neurospora* LSGs are shared between *N*. *crassa* and *N*. *discreta* with 26 showing no orthologs in *N*. *tetrasperma* (**S1B Fig**). Because of the special status of *N*. *crassa* as a model species, here we specifically investigate and report as LSGs those that are specific to *N*. *crassa* in this study. With more genomes in this fungal class being sequenced and annotated and more non-classified genes in the phylostratigraphy being analyzed, these numbers are expected to be slightly changed. Of the 670 *Neurospora* LSGs, 515 have at least one intron (average: ~2 introns, maximum: 8 introns in NCU07480). These LSGs encoded proteins ranging from 26 to 1310 amino acids (NCU05561 and NCU04852, respectively). The average LSG length (~192 amino acids) was significantly shorter than the average length of the non- *Neurospora* lineage-specific genes (non-LSG genes, ~528 amino acids).

### LSGs are aggregated in the telomere regions and clustered with the HET-domain genes

**Fig 2:**
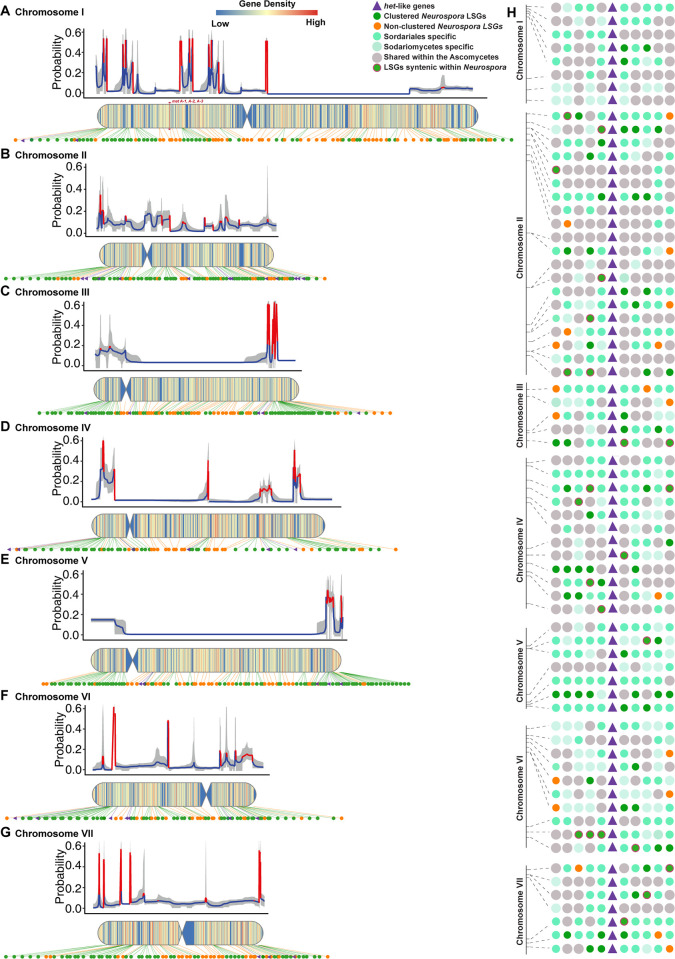
Identification of LSG and LSG-Het clusters in the *N*. *crassa* genome. Regionalized probability of the clustering status of a gene inferred using MACML, a window-free maximum-likelihood model-averaging approach, heat maps of clustering, and dot-plot of LSGs distributed across (**A**) chromosome I, (**B**) chromosome II, (**C**) chromosome III, (**D**) chromosome IV, (**E**) chromosome V, (**F**) chromosome VI, and (**G**) chromosome VII. Model-averaged profiles (blue: low clustering of LSGs vs non-LSGs; red: high clustering of LSGs vs non-LSGs; gray shading: 95% model uncertainty interval) quantify the window-free regionalized probability that a gene is an LSG. The heat maps quantify LSG-density across chromosomal windows of 10,000 base pairs. Dots located in accompanying chromosomal heat maps correspond to LSGs (clustered: green [*P* < 0.01], and non-clustered [*P* ≥ 0.01]: orange) and clustered HET-domain genes (purple triangles). (**H**) Lineage-specificity at four taxonomic depths (greener color intensity corresponds to lineage-specificity at successively lesser taxonomic depths: lineage specificity in *Neurospora* > Sordariales > Sordariomycetes > Ascomycetes; thin orange circle margin indicates synteny within *Neurospora*) of five neighbor genes on either side of 69 HET-domain genes (**S5 Table**) reported in the original genome-sequenced *N*. *crassa* strain [[FGSC2489; 31].

*Neurospora* LSGs are distributed in all seven chromosomes of the genome, with paralogs from duplicates often clustered together (**Fig 2**). Window-free maximum-likelihood model averaging of the gene-regionalized probability of *Neurospora* lineage-specific and HET-domain genes revealed that LSGs were clustered, with significant (*P* < 0.05) clustering on chromosomes I, II, III, IV, and V. Large LSGs clusters are typically aggregated toward the telomeres of each chromosome and frequently contain large non-coding spaces, especially in chromosomes I, III, IV, V, VI, and VII (**Fig 2A–2G and S2 Table**). Detailed clustering revealed with the Cluster Locator [29] tallied 67% of LSGs as present in clusters with a max-gap of five (48% with a max-gap of one, i.e., separated by one gene). About 30% of LSGs were in clusters with more than four genes (**Tables 1 and S3**), including six clusters hosting 9, 10, 16, and 23 genes.

**Table 1:** Lineage-specific and HET-domain genes are present in clusters in the *N*. *crassa* genome.

Cluster size^*c*^	LSG genes^*a*^			LSG and HET-domain genes^*b*^		
	# Clusters^*d*^	# Genes^*e*^	% Genes^*f*^	# Clusters (# HET)	# Genes (# HET)	% Genes
2	57	114	17.1%	63 (15)	126	17.1%
3	28	84	12.6%	28 (7)	84	11.4%
4	8	32	4.8%	12 (6)	48	6.5%
5	14	70	10.5%	13 (3)	65	8.8%
6	5	30	4.5%	11 (7)	66	8.9%
7	3	21	3.2%	2	14	1.9%
8	2	16	2.4%	2	16	2.2%
9	3	27	4.1%	1	9	1.2%
10–11	1	11	1.7%	2(2)	21	2.9%
16–18	1	16	2.4%	2(2)	35	4.8%
23	1	23	3.5%	1	23	3.1%
Total	123	444	66.8%	129 (42)	474 (42)	64.4%

^*a*^ 670 genes

^*b*^ 736 genes; 3 HET-domain genes are LSGs

^*c*^Cluster size is the number of genes in a given cluster

^*d*^# clusters is the number of clusters of the specified size

^*e*^# genes is the total number of genes in all of the clusters of the specified cluster size

^*f*^% genes in the genome.

A total of 68 *Neurospora* genes with HET domains (HET-domain genes) were identified and mapped to the *N*. *crassa* FGSC2489 strain [Fig 1 in reference 33]. Many of these HET-domain genes exhibited nonrandom distributions and were clustered near the end of the linkage groups, largely overlapping with clusters of LSGs (**Fig 2**). A previous study demonstrated that another HET-domain gene, NCU03125 (*het-C*), plays a role in vegetative incompatibility [30], but there are no LSGs clustered with this *het* gene. 42 of the 69 HET-domain genes were clustered with at least one LSG within a range of five genes (max-gap = 5), and 23 and 14 HET-domain genes were clustered with at least one LSG within a range of three (max-gap = 1) or two genes (max-gap = 0) separately (**Fig 2A–2G** and **Tables 1** and **S4**). In fact, many of HET-domain genes were only syntenic within *Neurospora* and very closely related species in the Sordariales, and most HET-domain genes were surrounded by *Neurospora* LSGs and comparatively “young” genes (**Fig 2H and S5 Table**).

### Many lineage-specific and HET-domain genes are dynamically expressed in response to developmental and environmental changes

**Fig 3:**
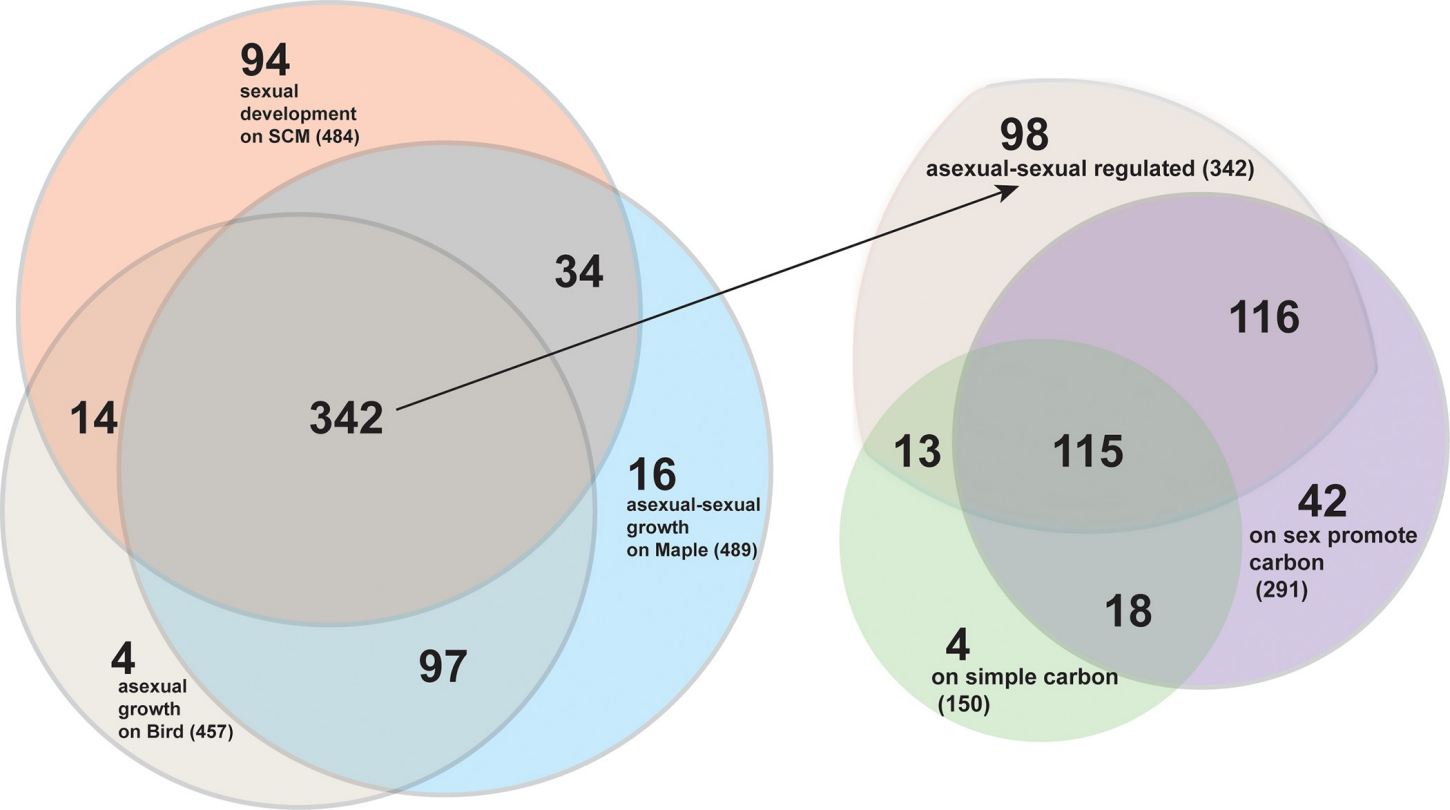
Differential expression of LSGs in *N*. *crassa* growth during three developmental processes [32,33] and on several media each supplying distinct carbon resources [34,35]. 342 LSGs were expressed at measurable levels in at least two stages in each of the three developmental processes (**S6 and S7 Tables**), including eight stages of sexual development on SCM (salmon pink, totaling 484 LSGs with measurable expression), four stages of asexual growth on BM (beige, totaling 457 LSGs with measurable expression) and four stages of asexual-sexual growth on MSM (light blue, totaling 489 LSGs with measurable expression). Among the 342 LSGs (arrow-linked), 231 were detectably expressed when cultured on either 2-furaldehyde furfural and/or 5-hydroxymethyl furfural (HMF), substrates that promote sexual development (purple, total 291 LSGs); and 128 were detectably expressed on sucrose and/or residues of at least one of five common crop straws (barley, corn, rice, soybean, and wheat; **S6 and S7 Tables**), substrates that support asexual growth and sporulation (light green, totaling 150 LSGs).

Genome-wide gene expression was measured in key stages of the *N*. *crassa* life cycle. Substantially different numbers of LSGs and non-LSGs were measurably expressed (**Figs 1**, **S2, and S3** and **Tables 2 and S6**). During sexual development on Synthetic Crossing Medium (SCM) and asexual growth on Bird Medium (BM), similar trends were observed for proportions of LSGs and non-LSG genes that exhibited measurable expression, but an increased proportion of LSGs were expressed during the early hyphal branching on Maple Sap Medium (MSM; **S2 Fig**). These media were designed to investigate specific environment-development associations in *N*. *crassa*, with SCM to induce and support sexual reproduction, BM only to support asexual reproduction, and MSM to support both sexual and asexual reproduction [32,33]. At one or more time points during perithecial development, 238 genes exhibited at least 5-fold (*P* < 0.05) expression changes during perithecial development, indicating potential roles in the regulation of sexual reproduction. Thirty-five genes exhibited no measurable expression in any sampled life history stages, suggesting either function only in unusual circumstances or mis-annotation as expressed genes (**S6 Table**). During conidial germination and early asexual growth, respectively, 95 and 137 LSGs exhibited at least 5-fold (*P* < 0.05) expression changes for cultures on BM and MSM, and 213 and 181LSGs were expressed during none or only one of the four sampled stages for cultures on BM or MS separately. Expression of 342 LSGs was detected in at least two sampled stages during sexual reproduction and during asexual growth on asexual specific BM and MSM (**Fig 3**).

**Table 2:** Measurable expression of lineage-specific, HET-domain, and other genes.

Experiments^1^	LSGs (670)		HET (66^2^)		other		Notes^3^ LSGs vs other (*P*)	GEO accession^4^
	expressed	Not expressed	expressed	Not expressed	expressed	Not expressed		
**25 C BM**	457	213 (82)	66	0	8668	326 (122)	***P*<0.01**	**GSE101412 [33]**
**25 C MSM**	489	181 (52)	66	0	8698	296 (113)	***P*<0.01**	**GSE101412 [33]**
**25 C SCM**	484	186 (49)	66	0	8764	230 (77)	***P*<0.01**	**GSE41484 [32]**
**37 C BM**	350	342 (84)	55	2 (1)	8616	710 (260)	***P*<0.01**	**GSE168995 [73]**
**Crop residue** ^**5**^	149	521	52	14	7497	1497	***P*<0.01**	**GSE60986 [34]**
**Furfural** ^**5**^	291 (31)	366 (32)	50 (2)	11 (4)	7763 (147)	968 (63)	***P*<0.01**; P<0.01; P<0.01	**In publication [35]**
**No carb** ^**5**^	184	486	52	14	7643	1351	***P*<0.01**	**GSE92848 [60]**
**Light** ^**6**^	224 (97)	381 (6)	25 (10)	31 (1)	7366 (381)	1701 (42)	***P*<0.01;** P<0.01; P<0.13	**GSE53534 [41]**
**Dark** ^**6**^	133 (6)	472 (97)	16 (1)	40 (10)	7027 (42)	2040 (381)	***P*<0.01;** P<0.13; P<0.01	**GSE53534 [41]**

^**1**^ In this multi-point gene expression data, only genes that were either expressed in at least two of the sampled points or not expressed at all in all sampled points were reported and considered as expressed or not expressed across the sampled process. For cultures sampled at multiple timepoints, some genes were expressed or not expressed only at one of the sampled points (tallied in parentheses).

^**2**^ Three predicted HET-domain genes—NCU03378 (*tol*), 07596, and 10839—are also identified as lineage-specific genes (LSGs).

^**3**^ Chi-square test *P* values regarding the proportion are provided for comparison between lineage-specific genes and non-HET non-lineage-specific genes that are color-coded in the rows.

^**4**^ Analyzed datasets with references.

^**5**^ The experiment compared *N*. *crassa* growing on media supplied with crop residue or furfural as carbon resources reporting expression of 9449 genes. Numbers of genes being expressed or silenced for cultures on the crop residue media, furfural media, and zero carbon medium, were reported. (Numbers in parentheses are the subset genes that are exclusivelyexpressed and exclusivelynotexpressed in cultures on furfural media). *P* values for three comparisons between LSGs and other genes in the genome are provided, including all expressed, exclusively expressed, and exclusively silenced genes. Significant *P* values (*P* < 0.05) are tabulated in boldface.

^**6**^ The light-exposure experiment tested dark and four light-exposure timepoints: 15-, 60-, 120-, and 240-minutes reporting expression of 9728 genes. The number of genes expressed or silenced in the light or in the dark are reported (numbers in parentheses are the subset genes that are exclusivelyexpressed or exclusivelynotexpressed). *P* values for three comparisons between LSGs and other genes in the genome were provided, including all expressed, exclusively expressed, and exclusively not expressed genes. Significant *P* values (*P* < 0.05) are tabulated in boldface.

Genomic gene expression was also assayed for *N*. *crassa* cultured on seven different carbon conditions, including absence of carbon, only glucose as a carbon source, and a complex crop-residue carbon source including components of barley, corn, rice, soybean, and wheat straws [34]. Expression of 464 LSGs was too low to be detected under any of these conditions. These LSGs likely do not play roles in carbon metabolism during vegetative growth (**S7 and S8 Tables**). Expression of 22 LSGs required that at least one type of carbon resource was present in the media, while expression of 56 other LSGs was only detected in the absence of carbon. Analysis of expression data collected from experiments investigating *N*. *crassa* tolerance to furfural [35] identified that 245, 239, 257, and 232 LSGs that exhibited measurable levels of expression in the simple carbon cultures, furfural, HMF treatments, and DMSO (the carbon blank control) (**S7 and S8 Tables**). Within the 291 genes expressed in either furfural or HMF cultures or both, 61 were not expressed in the wild-type condition. Furfural is derived from lignocellulosic biomass and enriched in a post-fire environment. *N*. *crassa* sexual spore germination can be induced by furfural presence [36,37]. Furfural also inhibits conidia germination. Compared with cultures under wild-type conditions, 12 LSGs exhibited a 3-fold or higher expression in response to furfural, with NCU09604, 07323, and 01153 upregulated 6- to 22-fold in furfural cultures.

**Fig 4:**
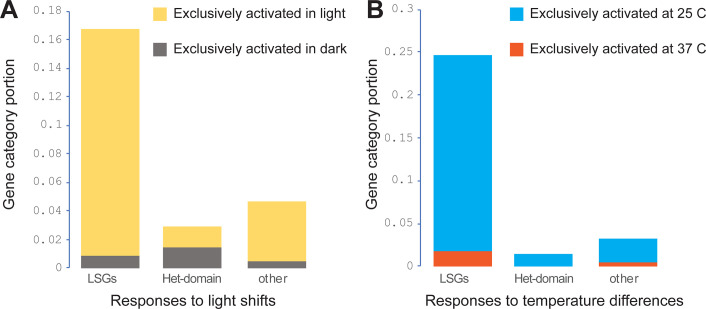
Genome-wide expression responses of LSGs, HET-domain, and other genes to shifting of culture environment. (**A**) Stacked proportions of 670 LSGs, 68 HET-domain, and other genes in the *N*. *crassa* genome, exhibited expression only in the dark (gray) or only in response to 15 to 240 minutes of light exposure (yellow). (**B**) Stacked proportions of 670 LSGs genes, 68 HET-domain genes, and other non-LSG genes in the *N*. *crassa* genome that are expressed across 4 stages of conidial germination and vegetative growth only at 25 C (blue) or 37 C (red) on Bird medium.

Environmental factors, including light and temperature that regulate fungal growth and development, also dramatically affect expression of LSGs (**S8 Table**). The *N*. *crassa* genome has genes encoding light sensors responding to different light spectrums, duration and intensities [38–43], and *Neurospora* LSGs exhibit sensitive responses to light conditions. When *N*. *crassa* cultures were exposed to light up to 4 h, genes were classified as short light responsive genes or long light responsive genes based on their expression profile changes [41]. Revisiting the previous data disclosed that out of 488 genes induced by light stimulus, 106 were LSGs (significantly enriched, *P* < 0.01), 59 of which were in the predicted clusters, including all genes in three 2-LSGs clusters, including cluster #25, 61, and 81 as well as three genes in a 4-gene cluster #127. Among 49 genes whose expression halted upon exposure to light, six were LSGs, including two genes NCU05052 and 05058 in a 3-gene cluster (**Fig 4A** and **S9 Table**). During conidial germination at a high temperature of 37 C on BM, expression of 270 LSGs was completely inhibited, a substantial enrichment (*P* < 0.01; a total of 941 out of 10592 genes exhibited no measurable expression). There were 148 LSGs exhibiting no measurable expression at 25 C and 37 C. There were 152 LSGs exhibiting detectable expression in cultures at 25 C but being turned off at 37 C, 100 of which were clustered LSGs, including 24 clusters with more than 2 genes that were turned off at 37 C. In contrast, 13 genes exhibited detectable expression in cultures at 37 C, but not at 25 C (**Fig 4B** and **S9 Table**). The LSG cluster #25 of NCU02144–02145 was the only LSG cluster that was silent in dark conditions or at 37 C.

Expression of HET-domain genes exhibited no clear patterns in response to environmental conditions or developmental stages **(Fig 4A and 4B and S8 and S9 Tables**). However, 11 HET-domain genes were not expressed in cultures on furfural or HMF, and 28 HET-domain genes were expressed neither in the dark nor during a shift from the dark to light for a duration of up to 2 h. Light stimulation is critical for phototropism and sexual development in *N*. *crassa* [44]. More genes were significantly up-regulated (5968 vs. 2935; *P* < 0.05) during the first branching of the germ tube on MSM, which supports both asexual and sexual development, than those on BM, which is specifically designed for promoting asexual reproduction and inhibiting sexual development. Accordingly, many more HET-domain genes were significantly up-regulated (46 vs. 15; *P* < 0.05) on MSM than on BM during that first branching stage.

### Some clustered lineage-specific and HET-domain genes exhibit coordinate expression

The majority of LSGs were clustered into physically linked groups—64% with *het* or HET-domain genes (**S4 Table**). Forty-two HET-domain genes clustered with at least one LSG, including HET-domain LSGs NCU03378, 07596, and 10839. Other than these three HET-domain LSGs, all clustered HET-domain genes exhibited measurable expression in at least three out of the four sampled stages in conidia germination as well as six out of eight stages sampled during sexual development.

**Fig 5:**
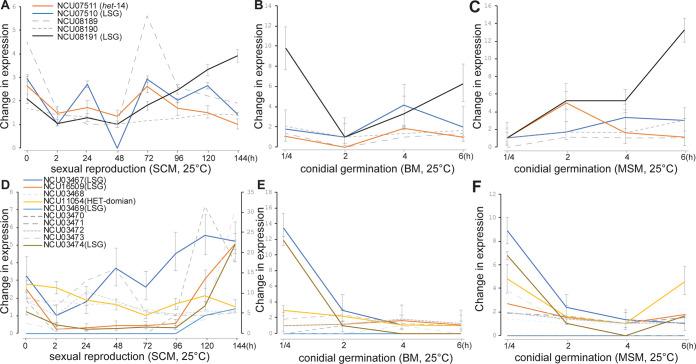
Expression profiles of two LSG-*het* gene clusters across asexual and sexual growth in *N*. *crassa*. expression profiles are plotted for LSG and HET-domain genes clustered with *het-14* (color coded, with 95% credible intervals)—as well as the non-LSG genes NCU08189 and NCU08910 located within the cluster (grey dashed)—cultured through (**A**) eight stages of sexual development on synthetic crossing medium, (**B**) four stages of conidial germination and asexual growth on Bird medium, and (**C**) four stages of conidial germination and asexual growth on maple sap medium. Expression profiles for genes clustered with *het-14* (color coded, with 95% credible intervals), including the five non-LSG genes NCU03468, 03470, 03471, 03472, and 03473 (grey dashed) within the cluster, across (**D**) eight stages of sexual development on synthetic crossing medium, (**E**) four stages of conidial germination and asexual growth on Bird medium, and (**F**) four stages of conidial germination and asexual growth on maple sap medium.

Coordinated expression among genes within the clusters during *N*. *crassa* asexual and sexual growth and development was not common. Among 26 cases where gene-expression dynamics were highly coordinated (i.e., at least one of the pairwise correlation coefficients were greater than 0.5 among LSGs in the cluster; **S10 Table**) across several developmental stages, two are notable (**Fig 5**): one, cluster #69, is a physically linked set of LSGs that exhibited coordinate expression and included NCU07511, a HET-domain gene that was at one time annotated as *het-14* (FungiDB.org), along with two other LSGs (NCU07510 and 08191). Genes in the cluster exhibited highly coordinated expression during sexual development, despite a large non-coding sequence of over 15 kbp that separates LSGs NCU07511 and NCU07510 in that cluster. Another notable cluster where gene-expression dynamics were highly coordinated across several developmental stages is the cluster #117 of a HET-domain gene (NCU11054) and four LSGs (NCU03467, 03469, 03474, and 16509). Genes in the cluster #117 exhibited highly coordinated expression during asexual growth on MSM (**Fig 5D–5F**). Expression was also observed to be coordinated in a few other clusters, such as among the LSGs clustered with the HET-domain genes NCU07335, 10142, and 16851 during conidial germination and asexual growth on BM and on MSM (**S4 Fig**): three clusters exhibited coordinated expression across sexual development (**S4A–S4C Fig**), eight exhibited coordinated expression across conidial germination and asexual growth on Bird medium (**S4D–S4K Fig**), and 15 exhibited coordinated expression across conidial germination and asexual growth on maple sap medium (**S4L–S4Z Fig**).

Two of the three LSGs clusters, including the cluster #24 (**S4A Fig**) and the cluster #131 (**S4C Fig**), exhibited coordinated expression among the genes within each cluster during sexual development, and the coordinate expression was observed during the early stages of sexual development before meiosis (about 48–72 h after crossing). Of the eight clusters, expression during asexual growth on BM was coordinately down-regulated in seven of them (**S4D–S4K Fig**). The exception was cluster #50 of HET-domain NCU07335 and LSG 07336; expression of these genes was up-regulated during sexual development (**S4I Fig**). Coordinate expression during asexual growth on MSM exhibited an opposite pattern: 12 out of 15 clusters exhibited up-regulated expression patterns toward the extension of the first hyphal branch (**S4L–S4Z Fig**), including two LSG-*het* clusters: the cluster #50 (**S4S Fig**) and the cluster #51 of NCU16851 (HET-domain), 07316, 07317, and 07323 (**S4T Fig**. In fact, coordinate expression was not only observed between lineage-specific and HET-domain genes that were clustered together, but also observed between LSGs in the clusters and neighboring non-LSGs (**S10 Table**). Furthermore, genes in cluster #112 exhibited no measurable expression in least at three out of the four sampled stages across conidial germination and across asexual growth, and genes in cluster #172 exhibited no measurable expression in at least six out of eight sampled stages in sexual development in *N*. *crassa*.

### Cell communication transcription factors affect expression of lineage-specific and HET-domain genes

**Fig 6:**
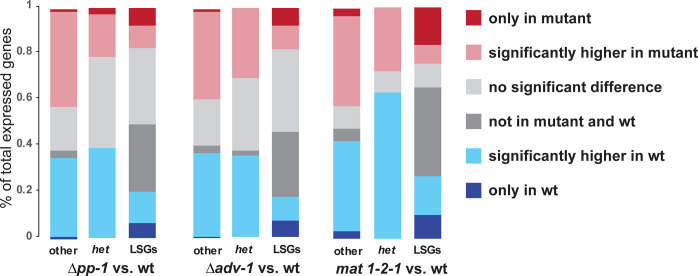
Impacts of transcription factor (TF) deletions on expression of HET-domain and LSGs within the *N*. *crassa* genome. Comparatively larger portions of LSGs are expressed exclusively in the mutants (dark red) or in the wildtype strains (dark blue). Expression profiles were classified in five categories (dark red: only measurable expression in mutant; light red: higher in mutant with *P* < 0.05; light grey: no significant difference with *P* ≥ 0.05; dark grey: not measurable in either mutant or wildtype; light blue: significantly higher in wildtype with *P* < 0.05; dark blue: only present in wildtype;), and comparative portions of each category in HET-domain genes, LSGs, and other genes in the genome were reported. Data from Fischer et al. (2018) were reanalyzed to assess the impacts of *pp-1* and *adv-1* knockout mutants.

The transcription factors *adv-1* and *pp-1* play multiple roles in asexual and sexual development, cell growth and fusion, and cell communication in *N*. *crassa* and closely related fungi [45–49]. Functions and regulatory networks involving *adv-1* and *pp-1* were systematically investigated [49], and in that study 155 genes were identified that were likely positively regulated by both transcription factors, including two HET-domain genes (NCU03494 and 09954) and one LSG (NCU17044). We reanalyzed the RNA sequencing data collected during conidial germination from knockout mutants of *adv-1* and *pp-1* and from wild type strain [49]. In general, knocking out the two transcription factors had a substantial impact on the activities of LSGs (**Figs 6 and S5 and Tables 3, S11 and S12**). Unlike HET-domain genes and other non-LSG genes, a significantly large portion of 173 LSGs exhibited no expression in the knockout mutants and the wild-type strain, including 196 not expressed in both *Δpp-1* and the wild type, and 195 not expressed in both *Δadv-1* and wild type. At the same time, significant numbers of LSGs were turned on or off by the mutations to the two transcription factors. Namely, the same number of 44 LSGs that were expressed in wild type were inactivated in the mutant strains *Δpp-1* or *Δadv-1*, and 23 LSGs were inactivated in both the mutant strains *Δpp-1* and *Δadv-1*. Among the 23 LSGs, 20 were within the predicted LSG-het clusters, including NCU04700 and 04710 that clustered with HET-domain gene NCU04694 and three core genes NCU08822, 08829, and 08830 in a six-gene cluster. At the same time, expression of significant numbers of LSGs (53 and 54 separately) went from undetectable to detectable in the *Δpp-1* or *Δadv-1* knockout strains, with expression of 31 LSGs becoming detectable in both knockout mutants. However, only 14 out of the 31 newly detectably expressed genes were clustered LSGs. Therefore, knocking out these two transcription factors did not substantially increase the expression level of genes in the LSG-HET domain gene clusters.

**Table 3:** Transcriptomic analyses of *N*. *crassa* responses to genetic manipulation.

Experiments^1^	LSGs (670)		HET (66^2^)			Other (8994)		Notes^3^ LSGs vs other (*P*)	GEO accession^4^
	expressed	Not expressed	expressed	Not expressed	expressed		Not expressed		
** *Δpp-1***	89 (106)	240 (249)	9 (21)	0 (2)	3582(2921)		400(457)	P<0.01(P<0.01); ***P<0*.*01*(*P<0*.*01*)**	**In publication [48]**
** *Δadv-1***	93 (90)	239 (249)	16 (21)	2 (2)	3207(3055)		425(457)	P<0.01(P<0.01); ***P<0*.*01*(*P<0*.*01*)**	**In publication [48]**
***mat a (1-2-1)***	155 (169)	70 (366)	17 (41)	0 (0)	3957(3966)		898(913)	P<0.01(P<0.01); *P>0*.*01***(*P<0*.*01*)**	**GSE199259 (this study)**
***Δpp-1* & *Δadv-1*** ^**5**^	49 (53)	196 (249)	12 (16)	0 (2)	1989(1685)		303(457)	P<0.01(P<0.01); ***P<0*.*01*(*P<0*.*01*)**	
***Δpp-1*& *mat a*** ^**5**^	26 (35)	202 (206)	2 (11)	0 (0)	1645(1236)		237(310)	P<0.01(P<0.01); ***P<0*.*01*(*P<0*.*01*)**	
***Mat a* & *Δadv-1*** ^**5**^	32 (22)	197 (206)	5 (12)	0 (0)	1524(1384)		259(310)	P<0.01(P<0.01); ***P<0*.*01*(*P<0*.*01*)**	

^**1**^ Comparisons were performed between mutant and wild type. Expressed genes refer to those that were well measured in each experiment. Numbers of genes with significantlyhigher (or, in parentheses, significantlylower) expression levels (*P* < 0.01, adjusted *P* < 0.05, in the knockouts or the mutant were provided. Silenced genes refer to those without measurable expression, and numbers of silenced genes in mutant and in wild type (in the parentheses) were reported.

^**2**^ Three predicted HET-domain genes, NCU03378 (*tol*), 07596, and 10839, were also identified as LSGs.

^**3**^ Four Chi-square test *P* values for deviation between counts of LSGs and non-LSG genes from marginal expectations for genes exhibiting (1) significantly higher expression (red), (2) lower expression (blue), (3) silencing in the mutant, and (4) exclusively silencing (numbers in parentheses for silenced genes are the subset of genes that exclusively silenced in the mutants).

^4^ Analyzed datasets with references.

^**5**^ Numbers of genes exhibiting significantly different expression between the wild type and co-existed in both mutants listed in the row (not a double-gene mutant). Specific gene IDs are listed in **S12 Table**.

Twenty-seven HET-domain genes are expressed at higher level in the wildtype strain than in both mutants (41 for *Δpp-1*, and 35 for *Δadv-1*), including four HET-domain genes (NCU03494, 06583, 09037, and 09954) exhibited more than five-fold higher expression in the wild-type strain than in the *Δpp-1* and/or *Δadv-1* strains. However, NCU03494, 06583, and 09037 are HET-domain genes not clustered with any LSGs. For lineage-specific and HET-domain genes with well measured expression in mutants and wild type, many genes exhibited similar up- or down-regulation between the *Δpp-1* and *Δadv-1* compared with their expression in the wild type (**S5 Fig**).

*N*. *crassa* mating loci regulate crossing and sexual development, and opposite mating-type strains are ordinarily vegetatively heterokaryon-incompatible [50–53]. Transcriptome profiles were compared between six-day cultures of the wildtype strain and a mating locus *mat 1-2-1* mutant that has lost mating function (FGSC#4564, *mat a*[m1]s-3B cyh-1) on synthetic crossing medium (SCM). Of 9758 measured genes, a total of 836 genes exhibited undetectable expression only in either the mutant or wildtype (**Figs 6 and S5 and S11 and S12 Tables**), including 179 LSGs (109 expressed and 70 undetected in the *mat 1-2-1* mutant) and 657 non-LSG genes (336 expressed and 321 undetected in the *mat 1-2-1* mutant). For LSGs, lack of detectable expression occurring only in the wildtype or only in the mutant was significantly enriched (*P* < 0.00001, chi-squared test). Of the 109 LSGs that were exclusively expressed in the *mat 1-2-1* mutant, 71 were located within 55 predicted LSG-*het* clusters. Of the 70 LSGs that were only expressed in the wild type, 59 were located within 47 predicted LSG-*het* clusters. Only 17 clusters were common between the two groups, and larger clusters with more than three genes presented behaved differently between the *mat 1-2-1* mutant and wild type, with three genes of five-gene cluster #51 (NCU07306–07323) being inactivated in the mutant, while four genes of seven-gene cluster #124 (NCU05480–06949) and five genes of the nine-gene cluster #94 (MCU07144–07152) being activated in the mutant. Some two-gene clusters exhibited coordinated expression that was detectable only in the mutant or the wild type. There were 53 LSGs that were expressed at significantly higher (*P* < 0.05) levels in the mating type mutant, and 111 LSGs that were expressed at significantly higher levels in the wild type. However, the mutation in the mating locus exhibited limited impact on expression of HET-domain genes, with 18 HET-domain genes being expressed significantly higher in the mating locus mutant, and 41 HET-domain genes being expressed significantly higher in the wild type (**Figs 6 and S5**).

Binding-site enrichment analysis using CiiiDER [54] identified no significant enrichment of binding sites for *mat 1-2-1*, *adv-1*, or *pp-1* in the upstream 5000 bp of LSGs that exhibited activity divergence in the mutants, specifying LSGs that whose expression was unchanged between the mutant and wildtype as the background. Lineage-specific and HET-domain genes were not significantly enriched in genes that are potentially bound by *adv-1* or *pp-1*, based on data from DAP-seq [SRP133627 from 49]. However, knocking out a key transcription factor encoding gene, *ada-6* [55]—whose product regulates asexual and sexual growth—inhibited expression of 30 LSGs and promoted expression of 25 LSGs during conidiation and protoperithecial production. Therefore, a substantial number of LSGs are at least peripherally involved in those regulatory networks.

### Protein functional structure analyses

To investigate if HET-domain genes and LSGs coding proteins have any domain structures with predictable gene functions, the genes were searched against the EMBL-EBI’s alphaFold database [56,57] and an inclusive Pfam library [58]. For the AlphaFold analysis with all the 670 LSGs, 13 types of domains were identified in 15 genes (**S1 Table**), with two CCHC-type domain containing genes (NCU16906 and 17247) and two CVNH-domain containing genes (NCU08168 and 17086). It is worth mentioning that the AlphaFold search identified LSG NCU03378 as a TOL protein-coding gene. From the Pfam analysis, for the HET-domain genes cited here from a previously study [24], all but NCU10839, 03378, and 07596 were annotated to belong to HET or Het-c family, while two genes were also annotated to contain an ankyrin repeats (NCU09772) or a protein-kinase domain (NCU06583; **S13 Table**). On the other hand, only 11 LSGs in *Neurospora* were annotated with one or more Pfams (**S14 Table**), revealing no LSG-specific enrichments of known functional domains.

### KO phenotypes of lineage-specific genes

**Fig 7:**
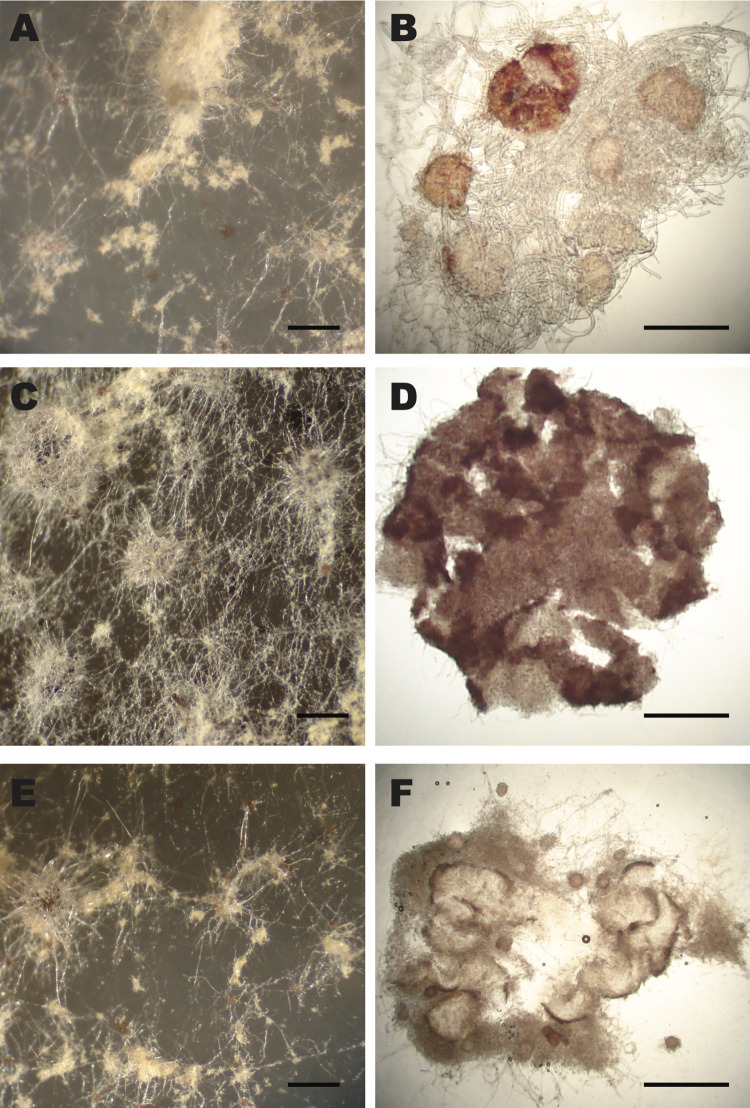
Knockout mutants of *Neurospora crassa* genes NCU00176, NCU00201, and NCU00529 produced arrested protoperithecia that failed to develop into perithecia and to produce sexual spores on SCM medium. Knockout cross ΔNCU00176 (FGSC12195 × 12196) exhibited (**A**) small protoperithecia (scale bar: 1 mm) and (**B**) squashed protoperithecia exhibiting an abortive ascogenous center (scale bar: 10 μm). Knockout cross ΔNCU00201 (FGSC18867 × 18868) exhibited (**C**) normal-sized protoperithecia (scale bar: 1 mm) with (**D**) abortive ascogenous centers (scale bar: 10 μm). Knockout cross ΔNCU00529 (FGSC13076 × 13077) exhibited (**E**) normal-sized protoperithecia with (**F**) abortive ascogenous centers.

In an examination of phenotypes of crosses of 367 available KO strains of the *Neurospora* LSGs to the KO of the opposite mating type (or the WT of the opposite mating type if the KO of the opposite mating type was not available), two *Neurospora* LSGs, NCU00176 and 00529, and one *Neurospora*-*Sordaria* LSG, NCU00201, showed a distinct knockout phenotype in sexual development (**Fig 7**). All these knockout mutants exhibited arrested development with the protoperithecia failing to develop into perithecia. Interestingly, both NCU00176 and NCU00201 were expressed at significantly higher levels in protoperithecia than in mycelia after crossing. Expression of NCU00529 was observed only in the late stage of conidial germination on maple medium. NCU00529, 00530, and 00531 are homologs, and NCU00529 and 00530 exhibited no expression in sexual development and conidial germination. No abnormal phenotypes were identified for NCU00531 knockout mutants (FGSC13078 and 13079), and no knockout mutants for NCU00530 are available. Cosegregation of hygromycin resistance and the observed phenotypes was used to confirm that the phenotypes of NCU00176, 00201, and 00529 were caused by the hygromycin-cassette-containing knockout lesion of the relevant genes. Normal perithecia were produced only on the wild type (FGSC2489, *mat A*) side of the crossing zone when co-cultured with *mat a* KO strains. Crosses between wild type and KO strains of NCU00176 and 00529 produced fewer normal perithecia than that between wild type and KO strain of NCU00201. All hygromycin- resistant cultures grown from these hygromycin resistant ascospores—12, 30, and 10 single-ascospore cultures for knockouts of NCU00176, 00201, and 00529 respectively—exhibited the phenotype of the knockout parent. Therefore, the observed phenotype was linked to hygromycin resistance, almost surely because of deletion of the target gene [46,59]. Knockouts of NCU00375, 00384, 00485, and 00491 (*Neurospora* LSGs) and NCU01623, 05395, 07618 (*Neurospora-Sordaria* LSGs) have been reported to exhibit minor phenotypic anomalies during asexual growth, especially at 37 C (Fungidb.org/fungidb). We examined knockout mutants of these genes and confirmed increased pigment production in NCU00485 and dense and slow growth in NCU00491 and 016223 at 37 C.

## Discussion

Here we have systematically investigated LSGs in *Neurospora* genomes to determine their location and organization on chromosomes, as well as their potential biological and ecological roles. Using genomic phylostratigraphy and reciprocal BLAST searches, we identified 670 LSGs that are only shared within *Neurospora* species. More than 63% of these 670 LSGs reside in clusters of 2–21 LSGs, interspersed with HET-domain putative allorecognition genes. Many of the larger clusters are aggregated near the telomeres of the seven chromosomes. A majority of *Neurospora* LSGs are actively regulated in response to carbon sources, light, and temperature changes that promote sexual or asexual reproduction. *Neurospora* strains with these 367 genes individually knocked out were phenotyped: in three cases, arrested protoperithecia were observed.

Transcriptomic profiles from environmental and genetic manipulations indicate that regulation of asexual and sexual reproduction may engage de *novo* elements in new roles early in their evolution. Specifically, we have provided evidence that this putatively preadaptive regulation is associated with key regulatory genes in cell-to-cell communication and with the shift of reproduction modes affected by carbon resources—a critical environmental factor for this post-fire fungus. In *N*. *crassa*, genetically identical hyphae fuse to establish and expand the colony during asexual growth. Hyphae of opposite mating type fuse to initiate sexual reproduction. Within both processes, a few HET-domain genes play critical roles. Many LSGs and their neighboring HET-domain genes were not identified as essential across any of the several phases of life history in the environments sampled in *N*. *crassa*. A substantial number of LSGs appear to be peripherally located in those regulatory networks, with patterns of expression consistent with regulatory roles, sparsely distributed enough to impact diverse regulatory pathways. Therefore, LSGs play roles as recently evolved fine regulators of response to environmental factors inducing understudied components of reproductive processes.

### Neurospora lineage-specific and HET-domain genes are tightly clustered

*Neurospora* lineage-specific and HET-domain genes exhibit several organizational features on chromosomes. These organizational features include LSG clustering, large non-coding spaces enclosed by flanking condensed LSG-encoding regions, frequent gene duplications, and proximity to telomeres. There are inherent challenges associated with tracing the molecular evolution of LSGs, Nevertheless, the origins of a few *Neurospora* LSGs were shown to be associated with gene duplications and chromosomal rearrangements enabled by the presence of long non-coding regions and repeat sequences [60].

Clustered genes that were derived from local rearrangement, along with occasional duplication and relocation, would easily functionally integrate into the extant gene expression regulatory network, due to their proximity to similarly regulated genes and ensuing common expression mechanisms and patterns. Indeed, several cases of coordinated expression across clustered lineage-specific and HET-domain genes were detected during *N*. *crassa* development in response to various environmental factors. However, LSG-HET-domain gene clusters and accompanying genes were syntenic mainly within very closely related taxa, and many LSGs exhibited no expression under diverse laboratory settings including a range of nutrient conditions and developmental stages. Interestingly, regulatory action via heterochromatic interactions via intra- and inter-telomeric contacts were reported as common in *N*. *crassa* [61], providing a means for *cis-* and *trans-*regulation at these LSG-enriched regions of the chromosome. It is possible that integration of LSG-HET-domain gene clusters into the extant regulatory systems modifies, temporally and spatially, the original functions that are known only under common growth conditions. Therefore, experiments conducted in alternate growth conditions, cultured with rare, specific, natural nutrient types, and at understudied stages of the life cycles should be conducted.

In the genomes of microbial eukaryotes, including yeast and microbial pathogens, telomeres and subtelomeric regions are characterized with tandem repeats and have been previously reported to feature gene clusters or gene families that have roles in adaptation to specific niches [62–64]. A previous study characterizing chromosome ends in *N*. *crassa* demonstrated that highly AT-rich sequences in the telomeres are likely products of the Repeat-Induced Point mutation (RIP) and that subtelomeric elements common in other fungi are absent in *N*. *crassa* [65]. RIP introduces high mutation rates and causes the deleterious consequences of repeated genomic regions due to gene duplication or transposable elements. Nevertheless, about 50% of unlinked duplications (due to chromosomal rearrangement) escape RIP in *Neurospora* [66]. Telomere repeats are required for H3K27 methylation, which would repress the transcription activities and functionally silent genes in these regions [67]. More importantly, the telomeric regions have potential significance in niche adaptation and probably harbor hotspots for novel sequences due to abrupt sequence divergence involving repeats [65]. Many genes with an annotated HET-domain also locate near the ends of *N*. *crassa* chromosomes [24], and 42 of the 69 HET-domain proteins—some of which are known to promote heterokaryon incompatibility—are actually clustered with at least one *Neurospora* LSG. In addition, synteny maps in the genome of *N*. *crassa* provide evidence that regions neighboring HET-domain genes are abundant with “young” genes—including but not exclusively LSGs. Many of these neighboring non-LSG genes have homologs in lineages outside of closely related species in the Sordariales, and are syntenic within Sordariomycetes. Such co-location of lineage-specific and HET-domain genes calls for investigation of possible co-functions or co-regulations in niche adaptation, which are directly related to reproduction successes. Expression profiles of clusters of lineage-specific and HET-domain genes were further examined for possible functional coordination. Most LSGs were not active in sampled stages in the *N*. *crassa* life cycle. However, a few LSG-HET-domain clusters exhibited coordinate expression during early sexual development as well as during active hyphal tip growth on the maple sap medium that supports both asexual and sexual development. Therefore, investigation of gene activities in the telomeric and sub-telomeric regions during mycelium development to sexual development will likely shed light on associations between these two functional groups in pre-mating processes and early sexual development in *Neurospora*.

Regulatory coordination between lineage-specific and HET-domain genes may be associated with the shift from asexual reproduction to sexual reproduction. Indeed, three LSGs—NCU03378, 07596, and 10839—were annotated with a HET-domain. NCU03378 was annotated as *tol* [tolerant, 51], and shared several conserved sequence regions with *het*-6 (NCU04453). *Het-6* promotes heterokaryon incompatibility in the *N*. *crassa* population [68] and was once identified as *tol* [69,70]. Functional associations among some HET-domain genes and LSGs within the LSG-*het* clusters were supported by coordinated expression regulation during asexual and sexual development in *N*. *crassa*. However, unlike the HET-domain genes which were all expressed, many LSGs were not measurably expressed across the sampled stages of the *N*. *crassa* life cycle. Clustered lineage-specific and HET-domain genes could be involved in consonant developmental processes and yet may not be consistently synchronized in expression; substantial co-regulation of genes with low expression dynamism may occur in unexamined stages of the *N*. *crassa* life cycle. In addition, functional roles of most HET-domain genes are barely known or fully investigated, except for a few *het* genes, even for the model fungus *N*. *crassa*. Further investigation of HET-domain genes could reveal functional associations between lineage-specific and HET-domain genes that are directly or tangentially related to cell-to-cell communication and, more broadly, reveal evolutionary opportunities and functional constraints related to *de novo* niche adaptation.

### Neurospora lineage-specific genes play roles in response to key regulatory environmental factors

LSGs frequently have not yet acquired any functional annotation, mainly due to the lack of homologous references to well-studied genomes wherein molecular genetic analysis has revealed function. To determine the functional roles of *Neurospora* LSGs, we revisited recent high-quality transcriptomics studies on *N*. *crassa*, covering almost all morphological stages in the *N*. *crassa* life cycle, cultures under different conditions and carbon or nitrogen resources, light exposure, temperature change, as well as knockout mutants of key regulatory genes [32–35,41,55,71–76]. We observed a significant enrichment of clustering of lineage-specific and HET-domain genes and, within some of those clusters, highly coordinated regulation in response to carbon resources, light, and temperature conditions. A substantial subset of the LSGs were actively responsive to differences in carbon source and temperature, and changes in exposure to light, providing evidence that some LSGs and clustered HET-domain genes are associated with adaptation to environmental factors that are critical indicators of successful fungal asexual and sexual growth and reproduction.

Three hundred forty-two LSGs that were actively expressed during sexual development were also actively regulated in both asexual growth on two different media: Bird medium, which supports only asexual reproduction; and maple sap medium, which supports both asexual and sexual growth. Nearly two thirds of the 342 genes were actively regulated in samples collected from the media supplied with carbon resources that promote sexual growth of *N*. *crassa*, supporting their possible roles in sexual development and the asexual-sexual switch. In favor of their roles in sexual development in *N*. *crassa*, more than one third of the LSGs were actively regulated in the presence of the specific carbohydrates HMF and furfural—compounds that powerfully stimulate initiation of sexual reproduction. *N*. *crassa* has been shown to respond differentially to the two furans and to possess a high tolerance to furfural, which is present in its natural habitat [35]. It is conceivable that some of the LSGs that are uniquely expressed upon exposure to HMF or furfural could be further engineered to provide increased tolerance to atypical carbon resources for *N*. *crassa*, a trait of significant interest in the pursuit of robust biofuel production.

Putative *Neurospora* orphan genes, many of which have been confirmed as LSGs here, have previously been reported to be active at the hyphal tips without measurable expression in the center of an expanding colony, leading to a hypothesis that LSGs perform roles in environmental sensing and in interaction with microbes [77]. Synthesizing these findings with our own, LSGs appear to typically be associated with reproductive development and growth in response to environmental conditions—especially carbon resources, light and temperature. We observed that LSGs in predicted clusters coordinately respond to these environmental factors. Our results suggest that LSGs in *Neurospora* are an excellent system to study how de novo and fast-evolving genes contribute to the fine tuning of quantitative switches related to reproductive decision-making in the face of changing environments. The substantial efforts of the community of scientists performing research on *N*. *crassa* to promote its use as a genetic model, in part by creating a collection of genome-wide knockout strains as well as extensive transcriptomic and genomic data sets spanning its biology and development [11,16,17,31,42,48,61], will facilitate further investigation into the roles of LSGs in biological and development processes.

### A significant proportion of LSGs are regulated by key developmental transcription factors

Transcription factors such as *pp-1* and *adv-1* that play key roles in cellular communication during asexual growth and mating loci that regulate sexual crossing, have been previously reported [49,50,78]. Expression of LSGs was significantly affected by perturbations of these genes. From the previous transcriptomics data from knockout mutants of *pp-1* and *adv-1* and newly generated transcriptomics data from a mutant of *mat 1-2-1*, we observed that the expression of a significant portion of LSGs was affected by mutations in these transcription factors. Interestingly, over 95% of LSGs that were turned off in both *pp-1* and *adv-1* mutants belonged to predicted clusters, but less than 50% LSGs that were turned on in both mutants belonged to predicted clusters. The expression of six genes in the NCU07144–07152 LSGs cluster was actively regulated during sexual development, in knockout mutants of *adv-1* and *pp-1* that regulate cell communication with knockout phenotypes in sexual development [45,48], as well as in samples in the absence of carbon. Expression of some LSGs was also affected in knockout mutants of other regulatory genes, such as *ada-6* and *gul-1* that are critical for sexual and asexual development in *N*. *crassa*. The most dramatic impacts to the expression of LSGs were from the mutation in the mating locus, suggesting likely functional associations of the mating process and sexual development initiation for LSGs. However, no binding sites of transcription factors were observed enriched in the promote and up-stream sequences of LSGs being turned on or off by those factors, and coordinate expression regulation was only detected in a few LSG-*het* gene clusters. Instead of possible cis or trans regulations, an alternative explanation for the associations between the LSGs and transcription factors would be that the LSGs were coordinated with the sampled developmental stages when the transcription factors were actively engaged. For example, the LSGs exhibited different expression activities during hyphal branching on natural medium MSM, when cell-to-cell communication is regulated by *adv-1*. Therefore, success in further investigations will be aided by focusing on the epigenetics of the LSG-*het* gene clusters during specific periods of cell-to-cell communication.

### Knockout phenotypes suggested that few LSGs play essential roles in Neurospora development

Our phenotyping of available knockout mutants of 367 LSGs available from the Fungal Genetics Stock Center [79] yielded three genes which when knocked out of a wildtype strain exhibited a phenotype in sexual development. One of these three genes, NCU00529, forms a cluster in the subtelomere of chromosome I with its homologs NCU00530 and 00531. The other two genes, NCU00176 and 00201, are present on chromosome III. Consistent with their knockout phenotypes of abortive sexual development at protoperithecial stage, expression of these three genes peaked at the formation of protoperithecia. However, a phenotype in sexual development could be the result from a more systematic impact of the gene knockout; for instance, many transcription factors were reported to have impacts on various stages of *N*. *crassa* development [48]. Indeed, NCU00176 is likely involved in *gul-1* regulatory pathways, as expression of NCU00176—along with seven other *Neurospora* LSGs—was down-regulated in the *gul-1* knockout mutant [80]. The *gul-1* gene plays multiple roles in *N*. *crassa* hyphal morphology and development [81].

A recent study reported 40 biologically relevant clusters (BRCs) for 1168 *N*. *crassa* genes being phenotyped for 10 growth and developmental processes [82]. Eleven *Neurospora* and 31 *Neurospora-Sordaria* LSGs were included in this analysis. Interestingly, 4 out of the 11 *Neurospora* (*P* < 0.05) and 5 out of the 31 *Neurospora-Sordaria* LSGs were concentrated in one of the 40 BRCs (Cluster 4 of 81 genes) and generally exhibit no significant phenotypes. Explanations for the lack of apparent phenotypes non-exclusively include that these genes 1) were not investigated under the conditions where the phenotypes manifest; 2) were functionally non-essential and/or not fully integrated into the regulatory networks, and/or 3) were functionally redundant within clusters or with non-LSG paralogs.

### Lineage-specific and co-clustered HET-domain genes exhibit some coordinated responses to genetic and environmental manipulations that govern reproductive mode

Investigation of functional interactions between lineage-specific and HET-domain genes using standard transcriptomics approaches is challenging because the two gene groups may function in different developmental phases and under different environmental conditions. Nevertheless, we observed limited coordinated responses to environmental and genetic regulatory factors between lineage-specific and HET-domain genes. The co-location of lineage-specific and HET-domain genes suggests that they could be both functionally and evolutionarily linked. The expression of LSGs is significantly altered during the transition from asexual to sexual reproduction in *N*. *crassa*, in response to environmental factors such as carbon resources, light, and temperature that are critical in regulating reproduction modes in the fungus. A significant portion of LSGs were also regulated by or responsive presence and absence of cell-to-cell communication transcription factors and mating types. Functional roles of most HET-domain genes are not known. However, a few HET-domain genes play critical roles in allorecognition during asexual growth and in mating process during sexual development. Further investigation at the species and population levels is required to determine the evolutionary histories of lineage-specific and HET-domain genes, which are linked by their co-location in recent lineages, and to guide experiments for investigating possible evolved co-ordinations between the two gene groups.

### Conclusion

Lineage-specific genes (LSGs) lack evolutionary histories tracing their ancestral relations within the genomes of other lineages, and are often classified as orphan or de novo genes. In this study, 670 *Neurospora* LSGs are reported, most of which are located in aggregates adjacent to the telomeres and are clustered along with “HET-domain” genes. The proteins encoded by LSGs possess few to no known functional domains that would help to identify potential roles in *N*. *crassa* biology and developmental biology. Transcriptomic profiling under environmental manipulations and in genetic mutants, and gene knockouts for phenotypes suggested that a large number of LSGs that are actively regulated during both asexual and sexual reproduction in response to carbon-resource, light, and temperature-based environmental factors. Expression of these LSGs is significantly affected by perturbation of the genes *adv-1*, *pp-1*, and the mating locus that regulates hyphal communication and initiation of sexual reproduction in the fungus. These observations encouraged further investigation of the roles of clustered lineage-specific and HET-domain genes in ecology and reproduction regulation in *Neurospora*, especially with pan-genomic and pan-transcriptomic data covering diverse environmental conditions and genetic backgrounds within the fungal species.

## Materials and methods

### Mutant and culture conditions

Protoperithecia were sampled for *mat 1-2-1* mutant (FGSC#4564, *mat a*[m1]s-3B cyh-1). The experiments were performed with macroconidia, which were harvested from 5-day cultures on Bird medium (BM). 1 × 10^5^ spores were placed onto the surface of a cellophane-covered synthetic crossing medium (SCM) in Petri dishes (60 mm, Falcon, Ref. 351007). Dark-colored protoperithecia were abundantly ripen in 6 days after inoculation. Tissue samples were flash frozen in liquid nitrogen and stored at -80 C. Biological replicates included all tissues collected from multiple plates in one collection process. Three biological replicates were prepared for each sampled point.

### RNA isolation and transcriptome profiling, data acquisition and analysis

Total RNA was extracted from homogenized tissue with TRI REAGENT (Molecular Research Center) as in Clark et al. (2008) [83], and sample preparation and sequencing followed our previous works [33,84,85]. Briefly, mRNA was purified using Dynabeads oligo(dT) magnetic separation (Invitrogen). RNAseq Library Prep: mRNA was purified from approximately 200 ng of total RNA with oligo-dT beads and sheared by incubation at 94 C in the presence of Mg (Roche Kapa mRNA Hyper Prep Catalog # KR1352).

For the first-strand cDNA synthesis, tA-tailing was performed with dUTP to generate strand-specific sequencing libraries. Indexed libraries were quantified by qRT-PCR using a commercially available kit (Roche KAPA Biosystems Cat# KK4854). The quality of cDNA samples was verified with a bioanalyzer (Agilent Technologies 2100).

The cDNA samples were sequenced at the Yale Center for Genomics Analysis (YCGA). The libraries underwent 76-bp single-end sequencing using an Illumina NovaSeq 6000 (S4 flow cell) according to Illumina protocols. Adapter sequences, empty reads, and low-quality sequences were removed. Trimmed reads were aligned to the *N*. *crassa* OR74A v12 genome from the Broad Institute [86] using HISAT2 v2.1, indicating that reads correspond to the reverse complement of the transcripts and reporting alignments tailored for transcript assemblers. Alignments with a quality score below 20 were excluded from further analysis. Reads were counted for each gene with StringTie v1.3.3 and the Python script prepDE.py provided in the package. StringTie was limited to report reads that matched the reference annotation. Sequence data and experiment details were made available (GSE199259) at the GEO database (https://www.ncbi.nlm.nih.gov/geo/).

Statistical analysis of the sequenced cDNA tallies for each sample was performed with LOX v1.6 [87], ignoring raw reads that mapped ambiguously or to multiple loci.

### Identification and verification of Neurospora crassa lineage specific genes (LSGs)

We applied a two-step strategy to identify and verify *Neurospora* LSGs, including (1) a phylostratigraphic approach to reveal putative LSGs and (2) confirmation using BLAST against all genomes available at NCBI and fungal genomes at FungiDB. In the first step, we employed previously published genomic phylostratigraphy for the *N*. *crassa* genome that reported over 2000 *N*. *crassa* orphan genes, discovered by genomic comparisons versus *Chaetomium globosum*, *Ascrospora strigata*, *Saccharomyces cerevisiae*, *Phanerochaete chrysosporium*, *Drosophila melanogaster*, and *Arabidopsis thaliana* [20]. *N*. *crassa* orphan-gene status was determined via the Smith-Waterman pairwise similarity of protein-coding sequences [88,89]. Classifications of genes in *N*. *crassa* were constructed as mutually exclusive groups ranked in by their phylostratigraphy, including Euk/Prok-core, Dikarya-core, Ascomycota-core, Pezizomycotina-specific, *N*. *crassa*-orphans, and others [12,90]. Putative *Neurospora* LSGs were also compared with the newest annotation of the *N*. *crassa* genome. Consequently, the number of predicted *N*. *crassa* LSGs based on the representative genomes was narrowed down to 1872 genes.

Many fungal genomes were recently published due to the efforts of the 1000 fungal genome project launched by the DOE Joint Genome Institute [23]. These fungal genomes—especially those well-sampled among closely related species—make it possible to be confident that LSGs are likely the product of de novo gene evolution rather than birth-and-death processes [91]. To verify that the 1872 genes are not present in species that were not analyzed within the phylostratigraphic and previous BLAST analyses, we employed BLASTp and tBLASTx to search in the entire NCBI GenBank database specifying an *E*-value cutoff of 0.05. To utilize newer genome annotations that were available on GenBank, BLASTp and tBLASTx were used again in FungiDB with an *E*-value cutoff of 10. These searches included genomes closely related to the Neurospora genomes, including *Podospora*, *Pyricularia*, *Ophiostoma*, *Chaetomium*, and *Sordaria* species. Any gene with a hit that was not from *Neurospora* was removed. This analysis results in 670 genes herein termed *Neurospora* LSGs.

Due to observed duplication history behind *Neurospora* lineage-specific genes, we enforced a strict expect threshold that identified 670 genes that likely are unique in *Neurospora*. To understand how these 670 LSGs identified from *N*. *crassa* are shared among the *Neurospora* genomes, a 3-way reciprocal blastp and a tblastx with a PAM30 score matrix were used to search for homologous genes among the three *Neurospora* genomes, and an *E* value of 1 × 10^−10^ was used as a cutoff, and synteny among the orthologs shared within the three *Neurospora* genomes was further visually checked at the FungiDB. Species-specific genes in *N*. *tetrasperma* and *N*. *discreta* were not investigated in this study, except for some specific cases mentioned in the text. For uncertain recent duplications, we also relied on the ortholog group identification in FungiDB, which reported potential homologs in 286 fungal and fungus-like Oomycetes genomes, including genomes for 35 Sordariomycetes species closely related to *Neurospora*. When necessary, additional phylogenetic analyses using the sequences downloaded from the FungalDB homologs groups were pursued to identify lineage-specific genes in *Neurospora*.

### Expression and functional analyses LSGs

Genome-wide gene expression was investigated in *N*. *crassa* along multiple stages of its life cycle, including conidial germination on different media [33] and production of meiotic propagules (ascospores) on synthetic crossing medium [32]. Transcriptomic data of GSE41484 was reanalyzed with the latest annotation of the *N*. *crassa* genome. The tally for each sample was processed with LOX v1.6 [87] to analyze gene expression levels across all data points, which uses a Bayesian algorithm to amalgamate different types of datasets. Gene counts or RPKM reads were analyzed by LOX, reporting relative expression of each gene normalized to the lowest treatment, 95% confidence intervals for relative expression, and statistical significance of expression differences. *P* values were adjusted following the procedure of Benjamin et al [92,93]. To assess environmental impacts on expression of LSGs, recent available data on 37 C BM from this lab (GSE168995) and a 240-minute time course of asexual growth in response to darkness and light stimulation [41] were revisited. To assess possible roles of LSGs in metabolic regulation, transcriptomics data from mycelia exposed to 5 different carbon resources from crop residues [34] and from mycelia in response to non-preferred carbon sources such as furfural and 5-hydroxymethyl furfural [HMF; 35] were also examined separately. To assess the gene expression effects of mutations of transcription factors, including *adv-1*, *pp-1*, and *ada-6*, transcriptomics data from Fisher et al. (2018) and Sun et al. (2019) [49,55] were also analyzed with LOX v1.6 [87]. Coordinated expression among genes within the LSG clusters was first manually examined. Twenty-six LSG clusters and their neighbor genes were selected for computation of pairwise correlation coefficients using R. Pairs of genes exhibiting coordination coefficients higher than 0.5 were considered coordinately expressed. Genes of *N*. *crassa* were functionally annotated with Pfam-scan (ver. 1.6), and Pfams of lineage-specific and HET-domain genes were then examined for predictable functions based on functional annotations of specific domains available from GO database and previous studies.

### Clustering analyses of LSGs heterogeneous variation sites and profiling of historical selection

The chromosomal distribution and clustering of LSGs—as well as lineage-specific and HET-domain genes—were analyzed with Cluster Locator [29]. Cluster Locator requires a parameter (Max-Gap) that specifies the number of genes that can be “skipped” between genes that are considered to be part of a cluster. We set Max-Gap = 5, 1, and 0 (Max-Gap. Statistically significant clusters (*P* < 0.01) were reported. To provide a more continuous measure of gene clustering, a vector of 0s and 1s representing non-LSG genes and LSGs was generated as an input sequence for MACML, a powerful algorithm for profiling the clustering of discrete ordered data [94]. This algorithm calculates all likely models of linear clustering by partitioning the entire sequence into all possible clusters and subclusters, and all models are statistically evaluated for information-optimality via Akaike Information Criterion [95], ‘corrected’ Akaike Information criterion [96], or Bayesian Information Criterion [97]. In this study, weighted likelihoods were computed based on the conservative Bayesian Information Criterion for each model. LSG cluster probabilities were calculated as weighted averages of models, and ninety-five percent model uncertainty intervals were calculated by further analyses of the model distributions.

### Knockout strains and phenotype identification

Knockout strains for more than 9600 genes [48], including deletion cassettes for genes in either of the two mating types, were acquired from the Fungal Genetic Stock Center [FGSC: 79]. Identified *Neurospora* LSGs were examined for altered phenotypes during conidia germination on Bird Medium (BM) and sexual development on Synthetic Crossing Medium (SCM) from protoperithecium differentiation to ascospore release. Genotype *mat A* strains were assayed for phenotypes when available; otherwise, *mat a* strains were used. All available KO strains were phenotyped on BM and on SCM with three replicates. For each investigated strain, 3000–5000 conidia were plated onto 90 mm diameter plates and monitored, and crossing was conducted between opposite mating types. Three independent phenotyping experiments were performed with each knockout strain using stored conidia supplied by the FGSC. Following previous studies [46,59], cosegregation experiments were performed to ensure that the intended deletion of NCU00176, 00201, and 00529 is responsible for the conspicuous mutant phenotypes in sexual reproduction. A hygromycin resistance cassette at the location of the deletion mutation provides a selectable marker. To assess cosegregation, the *mat a* KO strains were crossed with a wild-type strain (FGSC2489 *mat A*). Up to thirty individual ascospore progenies were isolated from BM plates supplied with 200 ug/ml hygromycin. Their phenotypes were then examined when co-cultured with mat A KO strains on SCM. Cosegregation of hygromycin resistance and the observed phenotype constitutes evidence that the observed phenotype was a result of the deletion of the specified gene.

## Supporting information

S1 Fig Genome-wide genes and *Neurospora* LSGs compared within the three *Neurospora* species *N*. *crassa*, *N*. *tetrasperma*, and *N*. *discreta*. (**A**) Comparative genomic protein-coding gene content among *N*. *crassa*, *N*. *discreta* and *N*. *tetrasperma*, centering shared single-copy orthologs within the three species. (**B**) Some *Neurospora* LSGs in the *N*. *crassa* genome are shared within *N*. *tetrasperma* and *N*. *discreta* genomes.
(PDF)

S2 FigProportion of *Neurospora* LSGs (blue) and non-LSG genes (orange) that were expressed at sampling points from sexual development on SCM and conidial germination and growth on BM and MSM.(**A**) Sexual development from protoperithecia (starting stage) to mature perithecia at 144 h [32]. (**B**) Asexual growth from conidial germination to the first hyphal branching on Bird medium supporting only asexual development. (**C**) Asexual growth from conidial germination to the first hyphal branching on maple sap medium supporting both asexual and sexual reproduction [conidial germination; 33].(PDF)

S3 Fig Lineage-specific gene expression dynamics across sexual development from protoperithecia (starting stage) to mature perithecia at 144 h [32] and asexual growth from conidial germination to the first hyphal branching [33], on Bird medium supporting only asexual development, and on a maple sap medium supporting both asexual and sexual reproduction. Microscopic morphologies of *N*. *crassa* at the sampled developmental points were provided. Heatmap was generated using the ClustVis web tool. Comparative gene expression was displayed as colors ranging from up- (red) to down- (blue) regulated as shown in the key.
(PDF)

S4 Fig Expression profiles of 21 LSGs clusters and LSG-*het* gene clusters (Table S4) across asexual and sexual growth in *N*. *crassa*. Expression and 95% credible intervals for (**A–C**) genes in clusters 24, 37, and 131 during sexual development, (**D–K**) genes in clusters 121, 128, 133, 88, 62, 50, 51 and 8 during conidial germination and asexual growth on Bird medium, and (**L–Z**) genes in clusters 22, 24, 33, 117, 63, 121, 65, 50, 51, 125, 87, 1, 8, 109 and 110 during conidial germination and asexual growth on maple sap medium.
(PDF)

S5 Fig Heat maps of HET-domain gene and LSG expression in conidial germlings that are wild-type, that have had *pp-1* and *adv-1* deleted, compared to fertilized protoperithecia of mutants of *mat 1-2-1*. (**A**) Expression divergence of HET-domain genes in the three mutants *vs*. wild type. Expression levels sampled in crossing were scaled in relation to the wild-type germling expression. HET-domain genes without measurable expression in wild type were excluded. (**B**) Expression divergence of LSGs in the three mutants *vs*. wild type. Expression levels sampled in crossing were scaled in relation to the wild-type germling expression. and LSGs without measurable expression in wild type were excluded.
(PDF)

S1 Table Identification of *Neurospora* lineage-specific genes (LSGs). (XLSX)

S2 Table MACML (Model Averaging Clustering by Maximum Likelihood) results. (XLSX)

S3 Table Significant clusters (*P* < 0.05) predicted for LSGs using Cluster Locator with Max Gap set to be 5, 1 and 0. (XLSX)

S4 Table Significant clusters predicted for LSGs & *het*-like genes using Cluster Locator with Max Gap set to be 5, 1 and 0. (XLSX)

S5 Table FungiDB Phylogenetic synteny status for 69 HET-domain genes (highlighted in yellow) and their neighbor genes (for Fig 2H). (XLSX)

S6 Table Clusters of LSG and HET-domain genes on chromosomes and their relative expression across developmental timepoints of conidial germination cultures on Bird medium that induces only asexual growth and development, on maple medium that supports asexual development, sexual development, the asex-sex switch, and of sexual development on Synthetic Crossing medium. Gene expression is quantified in fold-change compared to the lowest expression across the developmental timecourse, which is set at 1. Expression that was too low to be measurable is reported as 0.
(XLSX)

S7 Table LSGs that are actively regulated in distinct developmental and carbon conditions (summarized in Fig 3). (XLSX)

S8 Table Relative gene expression levels across measurements for all well-measured genes reported in four publicly accessible datasets regarding *N*. *crassa* gene expression in distinct environmental settings. Gene expression fold-changes are normalized against the lowest expression of the gene in the experiment, which is set at a value of 1. Expression that was too low to be measurable was set to be 0.
(XLSX)

S9 Table LSGs and HET-domain genes exhibited divergent expression in response to light and temperature conditions. (XLSX)

S10 Table Correlation coefficients of expression for selected clusters of LSGs (red) and neighboring non-LSGs under distinct growth conditions as in S5 and S3 Figs. (XLSX)

S11 Table Relative gene expression in mutants of *mat 1-2-1*, *adv-1*, *pp-1*, and *ada-6*. (XLSX)

S12 Table LSGs and *het*-like genes that respond to transcription-factor knockouts Δ*pp-1* and Δ*adv-1* and a mutant mating locus. Significant expression is ascertained based on LOX *P* < 0.01 and adjusted LOX *P* < 0.05.
(XLSX)

S13 Table Protein families of *Neurospora* HET-domain genes. (XLSX)

S14 Table Protein families of *Neurospora* LSGs. (XLSX)
