# Supplementary figures and images for: Lineage-specific genes are clustered with HET-domain genes and respond to environmental and genetic manipulations regulating reproduction in Neurospora
Source: PLoS Genet. 2023 Nov 7;19(11):e1011019. doi: 10.1371/journal.pgen.1011019 (PMC10684091; doi:10.1371/journal.pgen.1011019)

**A****9948 genes/9043 ortholog groups***N. discreta*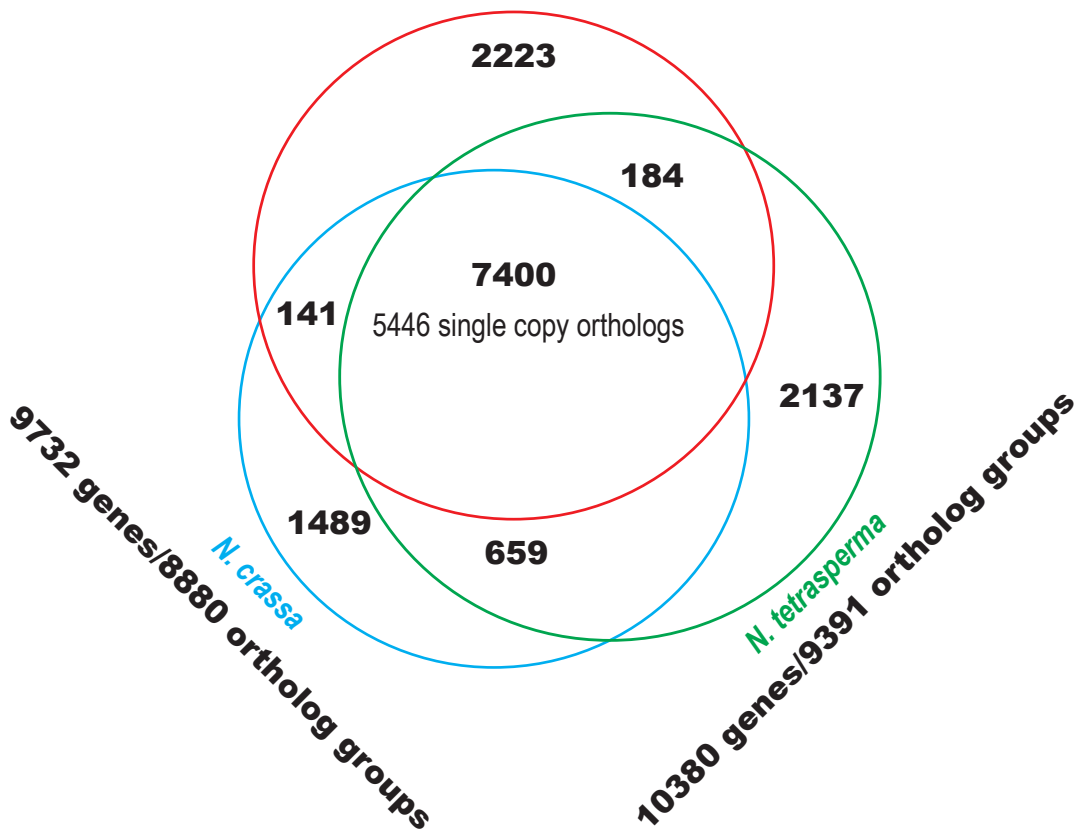**B*****N. crassa* LSGs shared  
with other *Neurospora* species**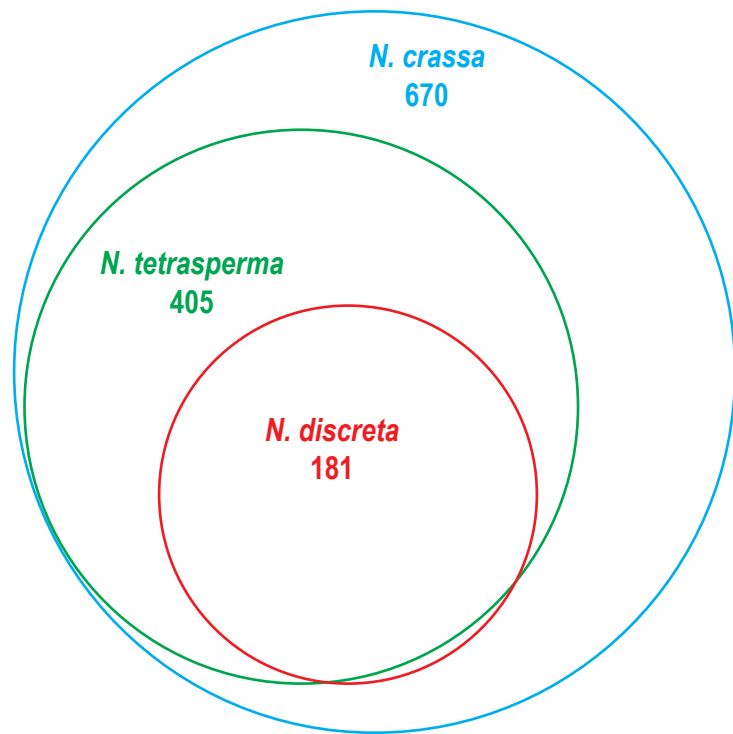

Supplement: S1 Fig — (A) Comparative genomic protein-coding gene content among N. crassa, N. discreta and N. tetrasperma, centering shared single-copy orthologs within the three species. (B) Some Neurospora LSGs in the N. crassa genome are shared within N. tetrasperma and N. discreta genomes. (PDF) [file pgen.1011019.s001.pdf]

**A**

Expressed non-LSG genes (%)

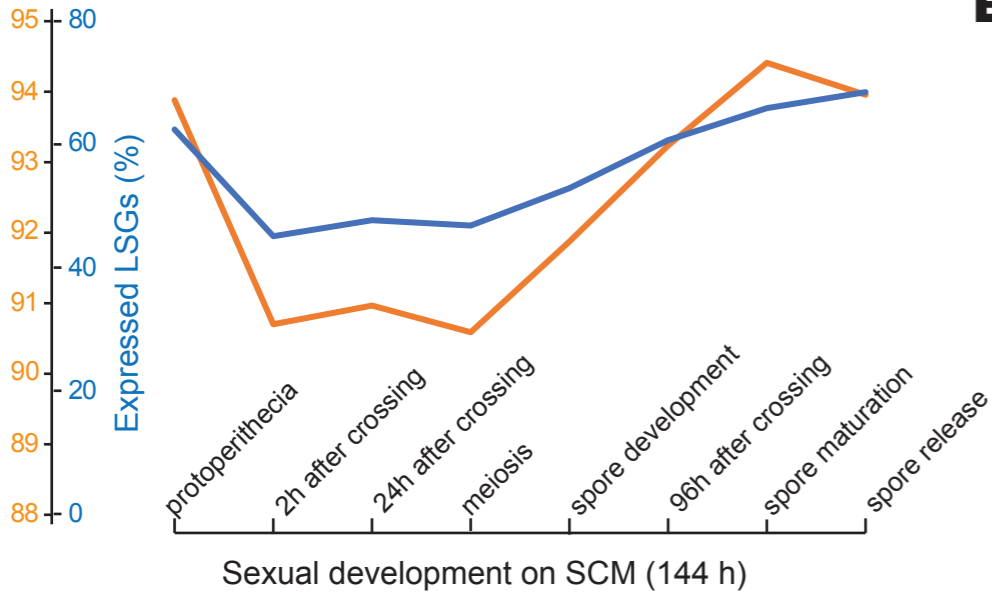**B**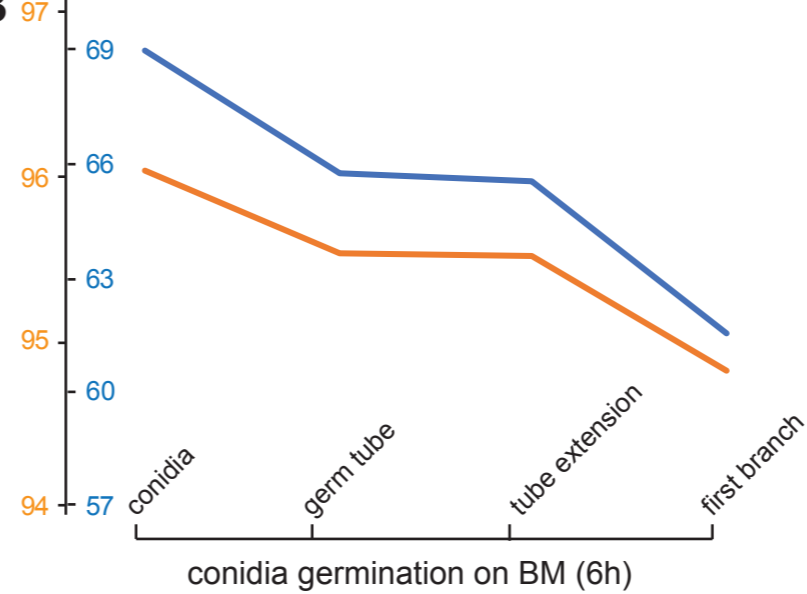**C**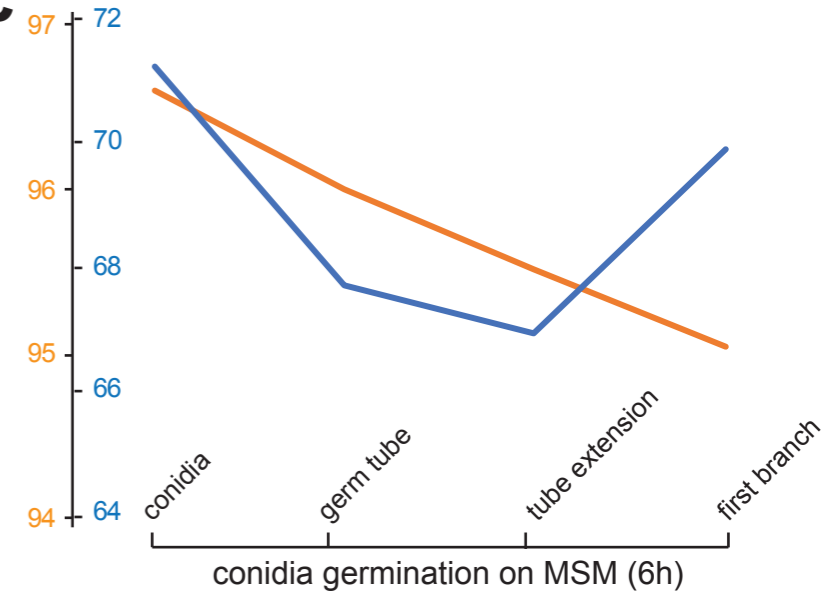

Supplement: S2 Fig — (A) Sexual development from protoperithecia (starting stage) to mature perithecia at 144 h [32]. (B) Asexual growth from conidial germination to the first hyphal branching on Bird medium supporting only asexual development. (C) Asexual growth from conidial germination to the first hyphal branching on maple sap medium supporting both asexual and sexual reproduction [conidial germination; 33]. (PDF) [file pgen.1011019.s002.pdf]

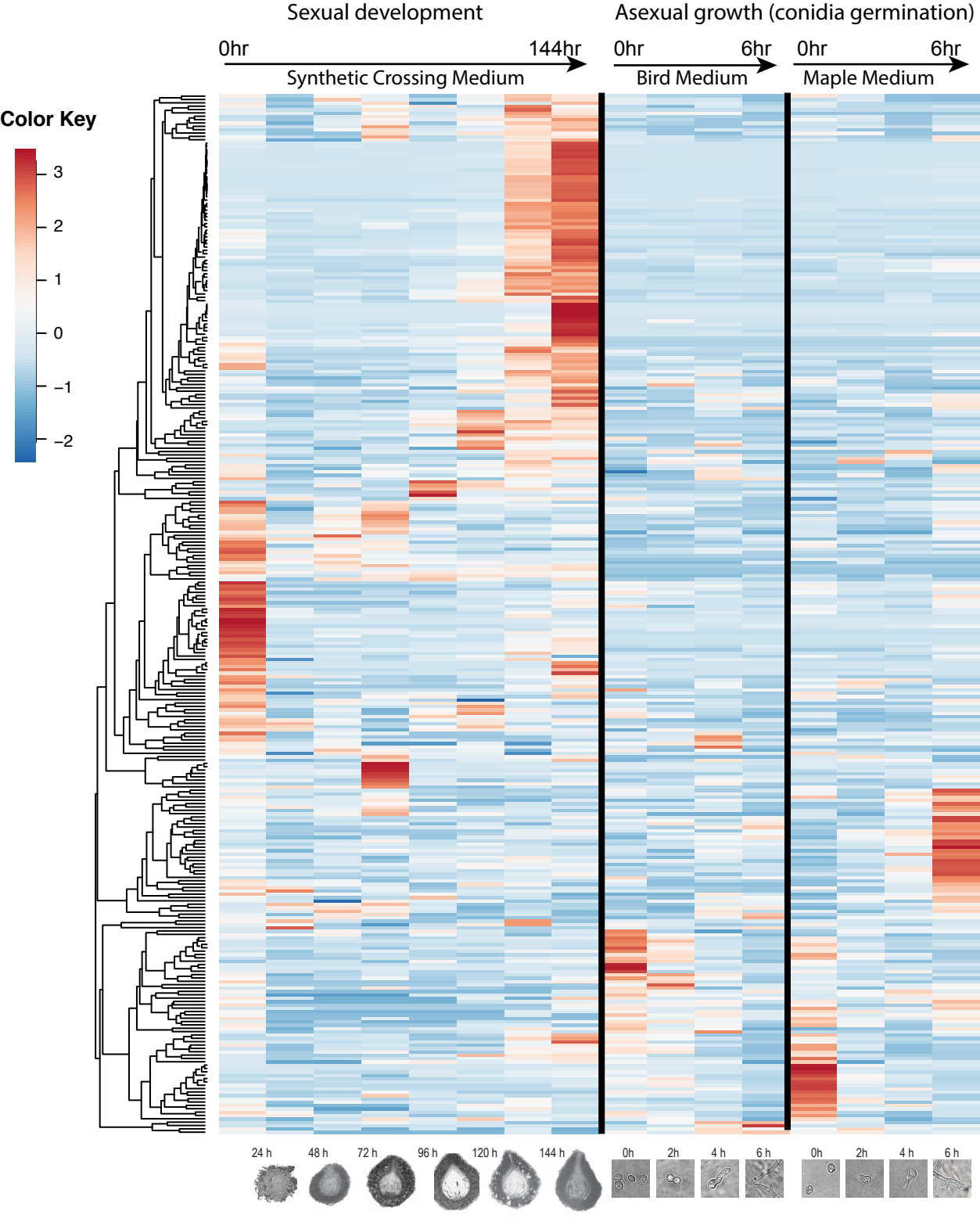

Supplement: S3 Fig — Microscopic morphologies of N. crassa at the sampled developmental points were provided. Heatmap was generated using the ClustVis web tool. Comparative gene expression was displayed as colors ranging from up- (red) to down- (blue) regulated as shown in the key. (PDF) [file pgen.1011019.s003.pdf]

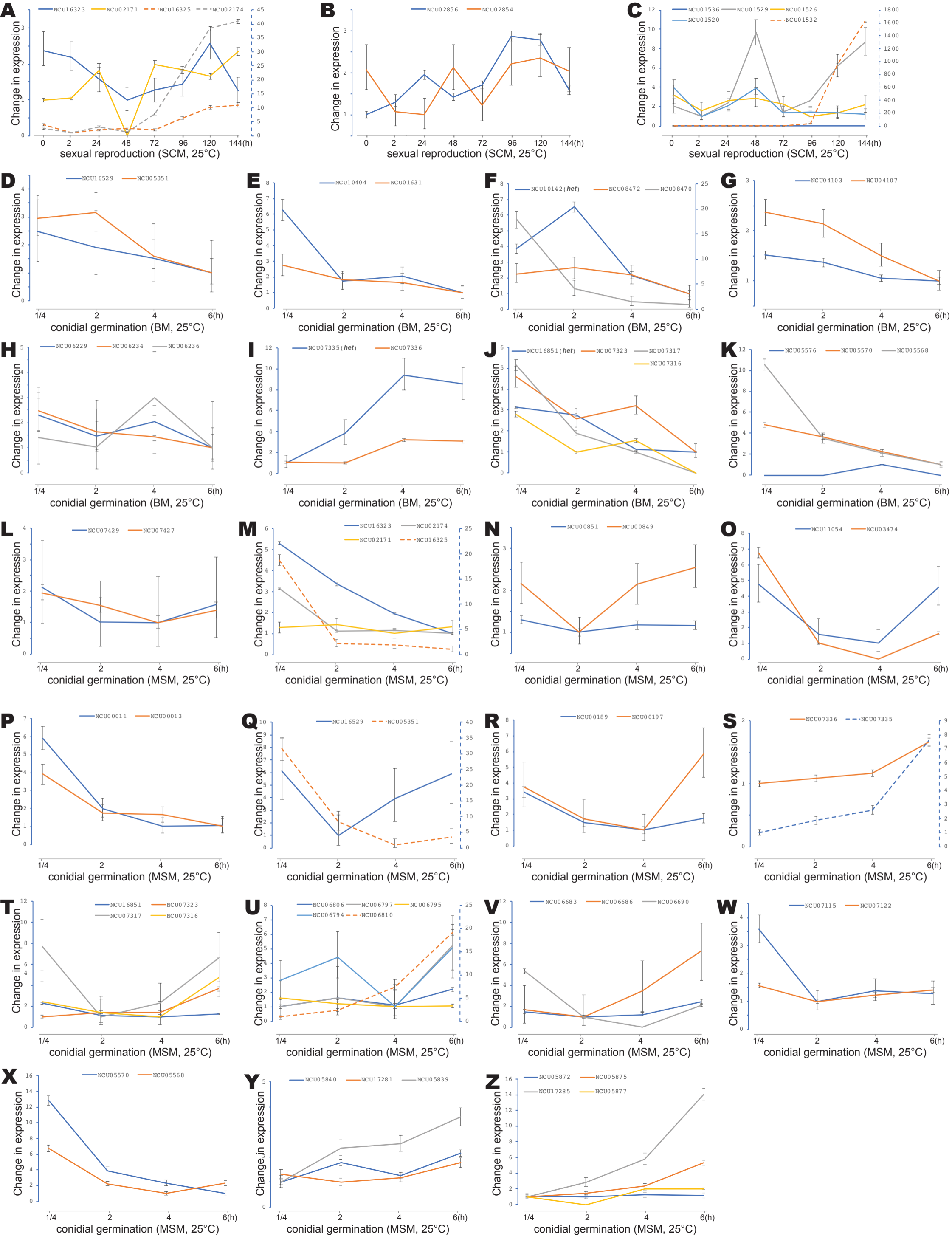

Supplement: S4 Fig — Expression profiles of 21 LSGs clusters and LSG-het gene clusters (Table S4) across asexual and sexual growth in N. crassa. Expression and 95% credible intervals for (A–C) genes in clusters 24, 37, and 131 during sexual development, (D–K) genes in clusters 121, 128, 133, 88, 62, 50, 51 and 8 during conidial germination and asexual growth on Bird medium, and (L–Z) genes in clusters 22, 24, 33, 117, 63, 121, 65, 50, 51, 125, 87, 1, 8, 109 and 110 during conidial germination and asexual growth on maple sap medium. (PDF) [file pgen.1011019.s004.pdf]

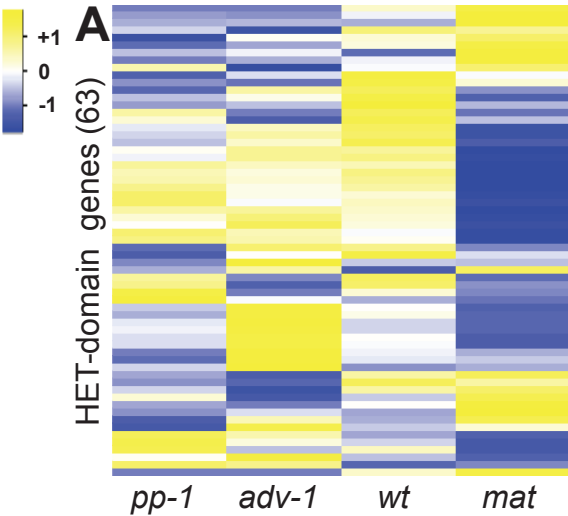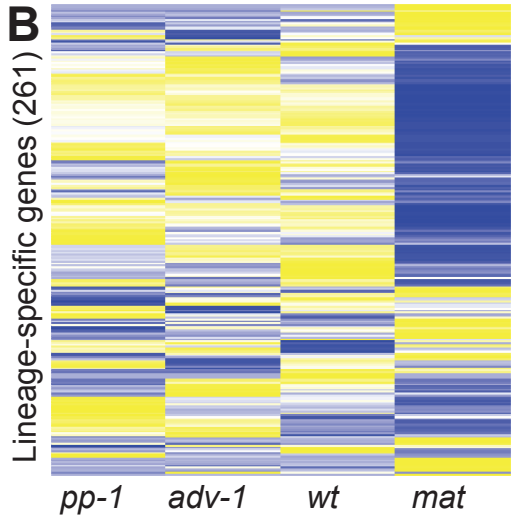

Supplement: S5 Fig — (A) Expression divergence of HET-domain genes in the three mutants vs. wild type. Expression levels sampled in crossing were scaled in relation to the wild-type germling expression. HET-domain genes without measurable expression in wild type were excluded. (B) Expression divergence of LSGs in the three mutants vs. wild type. Expression levels sampled in crossing were scaled in relation to the wild-type germling expression. and LSGs without measurable expression in wild type were excluded. (PDF) [file pgen.1011019.s005.pdf]
